# Bacteriophages suppress CRISPR–Cas immunity using RNA-based anti-CRISPRs

**DOI:** 10.1038/s41586-023-06612-5

**Published:** 2023-10-18

**Authors:** Sarah Camara-Wilpert, David Mayo-Muñoz, Jakob Russel, Robert D. Fagerlund, Jonas S. Madsen, Peter C. Fineran, Søren J. Sørensen, Rafael Pinilla-Redondo

**Affiliations:** 1 Section of Microbiology, University of Copenhagen, Copenhagen, Denmark; 2 Department of Microbiology and Immunology, University of Otago, Dunedin, New Zealand; 3 Genetics Otago, University of Otago, Dunedin, New Zealand; 4 Maurice Wilkins Centre for Molecular Biodiscovery, University of Otago, Dunedin, New Zealand; 5 Bioprotection Aotearoa, University of Otago, Dunedin, New Zealand

**Keywords:** Bacteriophages, Viral genetics, Small RNAs

## Abstract

Many bacteria use CRISPR–Cas systems to combat mobile genetic elements, such as bacteriophages and plasmids^1^. In turn, these invasive elements have evolved anti-CRISPR proteins to block host immunity^2,3^. Here we unveil a distinct type of CRISPR–Cas Inhibition strategy that is based on small non-coding RNA anti-CRISPRs (Racrs). Racrs mimic the repeats found in CRISPR arrays and are encoded in viral genomes as solitary repeat units^4^. We show that a prophage-encoded Racr strongly inhibits the type I-F CRISPR–Cas system by interacting specifically with Cas6f and Cas7f, resulting in the formation of an aberrant Cas subcomplex. We identified Racr candidates for almost all CRISPR–Cas types encoded by a diverse range of viruses and plasmids, often in the genetic context of other anti-CRISPR genes^5^. Functional testing of nine candidates spanning the two CRISPR–Cas classes confirmed their strong immune inhibitory function. Our results demonstrate that molecular mimicry of CRISPR repeats is a widespread anti-CRISPR strategy, which opens the door to potential biotechnological applications^6^.

## Abstract (web summary)

In response to bacterial CRISPR–Cas immunity, phages and plasmids have evolved small non-coding RNA anti-CRISPRs, known as Racrs, that sequester Cas proteins in abberrant complexes and thereby inhibit immunity.

## Main

Bacteriophages (phages) and other mobile genetic elements (MGEs) exert an immense selective pressure on bacteria, which in response have developed a broad arsenal of defence mechanisms^7,8^. Among these, CRISPR–Cas (clustered regularly interspaced short palindromic repeats–CRISPR-associated proteins) is a group of widespread RNA-guided adaptive immune systems that are classified into two broad classes, six types and numerous subtypes according to their genetic composition and interference mechanism^1^. The CRISPR–Cas immune response starts with the acquisition of short DNA fragments (protospacers) from invading MGEs. The protospacers are inserted as spacers between repeats in the CRISPR array to create a memory of the infection. Next, the CRISPR array is expressed as a long transcript that is processed into small, mature CRISPR RNAs (crRNAs), each carrying a spacer sequence flanked by part of the repeat. Finally, the interference complexes, composed of a crRNA and one (class 2) or more (class 1) Cas proteins, degrade the complementary nucleic-acid targets that are often found next to a short protospacer-adjacent motif (PAM)^1^. The specificity and programmability of the CRISPR–Cas machinery has led to the development of various biotechnological applications in genome editing, molecular diagnostics and more^9^.

In the evolutionary arms race with CRISPR–Cas, phages and other MGEs have evolved diverse strategies to block or circumvent immunity^10^. One widespread evasion mechanism uses protein-based CRISPR–Cas inhibitors called anti-CRISPRs (Acrs)^2,3^. So far, more than 100 Acr protein families have been identified that inhibit different stages of the CRISPR–Cas immune response, mainly by interacting directly with Cas proteins. For example, Acrs prevent crRNA loading, effector-complex formation, and target DNA binding and cleavage^2^. Notably, the discovery of these natural ‘off switches’ has presented new opportunities to control the activity of CRISPR–Cas technologies^6^.

A growing body of work highlights the frequent co-option of CRISPR–Cas systems and their components by diverse MGEs^4^. Some specific CRISPR–Cas associations with MGEs have been characterized in detail, including crRNA-guided transposition^11,12^, transcriptional repression^13^ and inter-viral^14^ and inter-plasmid^15,16^ conflicts, but others are poorly understood. An intriguing case is the bioinformatic identification in viral genomes of solitary repeat units (SRUs), which are often immediately downstream of a predicted promoter and not associated with *cas* genes^4^. Although the biological function of viral-encoded SRUs is not fully understood, their similarity to direct repeats in CRISPR loci led to speculation that they are involved in CRISPR–Cas inhibition. It has been suggested that they may interact with host Cas components or enable viral integration in CRISPR arrays^4,17^. Here we demonstrate that many SRUs function as RNA Acrs (Racrs) that bind to Cas proteins to interfere with the formation of canonical CRISPR-Cas effector complexes. We show that CRISPR repeat mimicry is a widespread immune-evasion strategy used by phages and plasmids that infect diverse prokaryotic taxa.

## A phage-derived SRU inhibits CRISPR–Cas

**Fig. 1:**
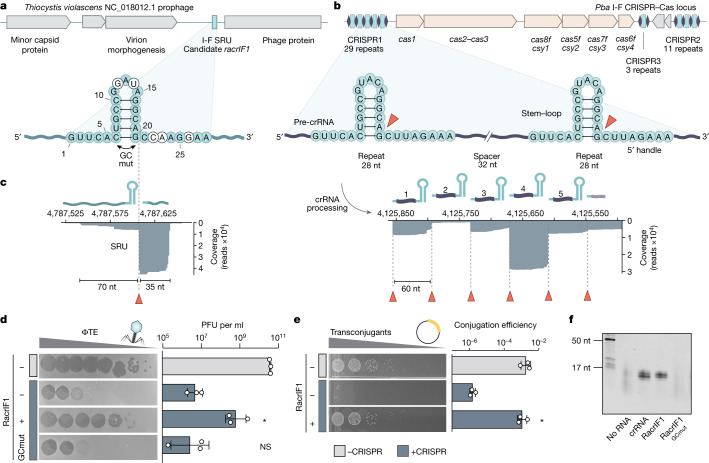
RacrIF1 displays anti-CRISPR activity. **a**, Schematic (top) of the region of the *T.* *violascens* prophage that encodes a type I-F SRU (PPOA865; light blue). The predicted secondary RNA structure of the I-F SRU (bottom) shows bases that differ (white) from those in the consensus direct repeat (light blue) of the *P.* *atrosepticum* (*Pba*) type I-F CRISPR arrays in **b**. The C6G/G20C mutation at the base of the I-F SRU stem applied in **d** is indicated (RacrIF1^GCmut^, GC mut). **b**, Schematic (top) of the type I-F CRISPR–Cas locus from *P.* *atrosepticum* strain SCRI1043 (ref. ^32^), and secondary RNA structure of the type I-F direct repeat (middle). Orange arrowheads show the Cas6f processing site. Bottom, small RNA-seq data mapping to a section of the CRISPR1 array. **c**, Small RNA-seq data from *P.* *atrosepticum* mapping to the I-F SRU and flanking regions. **d**, Plaque-forming units (PFU) per ml for ΦTE infecting *P.* *atrosepticum* non-targeting (–CRISPR, grey) or targeting (+CRISPR, blue) the phage, carrying either an empty vector control (–RacrIF1) or a plasmid encoding the type I-F SRU (+RacrIF1) or RacrIF1^GCmut^ (GCmut) expressed from the wild-type promoter. **e**, Conjugation efficiency of a type I-F targeted plasmid (+CRISPR, blue) into wild-type *P.* *atrosepticum* compared with an untargeted control (–CRISPR, grey), containing either a plasmid expressing RacrIF1 (+RacrIF1) from the P_BAD_ promoter or an empty vector control (–RacrIF1). Data in **d** and **e** represent *n* = 3 biological replicates plotted as the mean ± s.d., with pictures of one representative sample. Statistical significance was assessed using a one-way ANOVA test of +Racr samples compared with the –Racr +CRISPR control (*P ≤ 0.05; NS, not significant). **f**, RNA isolated after affinity purification and SEC (Extended Data Fig. 1h) of His_6_–Cas6f co-expressed with different RNA variants: type I-F crRNA, RacrIF1, RacrIF1^GCmut^ or an empty vector with no RNA as a control (for gel source data, see Supplementary Fig. 1). Source data

**Extended Data Fig. 1:**
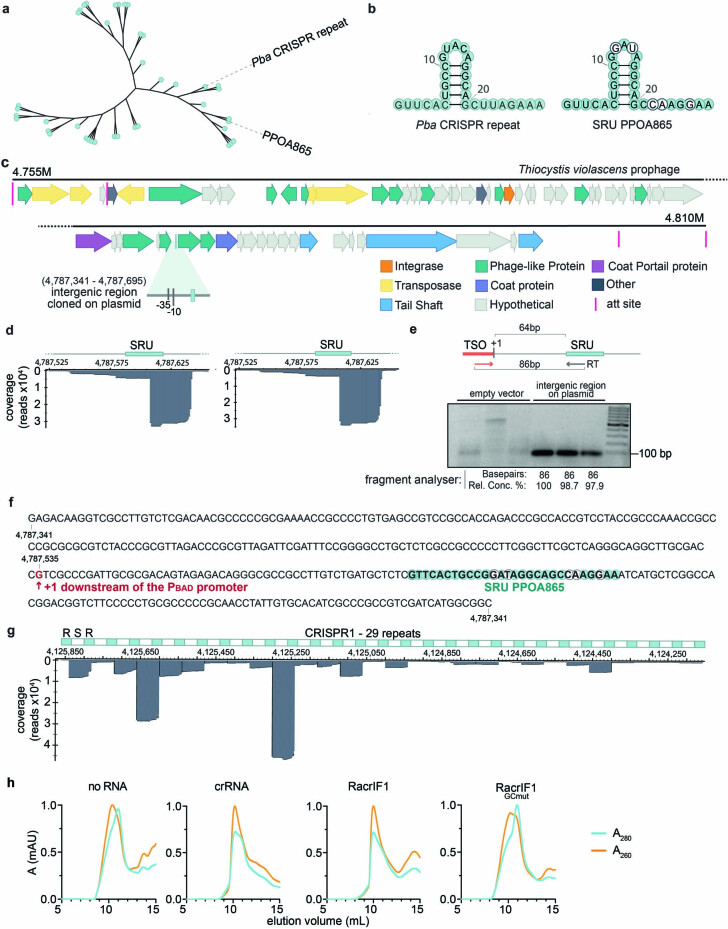
Genetic context and expression of RacrIF1. **a**, Phylogenetic tree of the I-F classified SRUs from Faure et al. (2019)^4^. with the *P. atrosepticum* (*Pba*) consensus repeat. **b**, Depiction of the secondary structures of I-F SRU PPOA865 and *Pba* CRISPR repeat. **c**, Schematic of the predicted prophage carrying the I-F SRU PPOA865, detailing the sequences cloned into expression vectors used for functional testing. **d**, Mapping of small RNA-seq data to the type I-F SRU and flanking regions encoded on a plasmid (pPF2845). Data shown are additional biological replicates of Fig. 1c. **e**, 5′ RACE analysis of the intergenic region cloned into an expression vector under wild-type promoter expression and empty vector control. The template switching oligo (TSO), complementary DNA (cDNA) sizes and relative abundance determined with the fragment analyser are indicated (for gel source data, see Supplementary Fig. 1). **f**, Depiction of the full length intergenic region cloned into RacrIF1 expression plasmid. The +1 transcription start site (TSS) used for P_BAD_ promoter expression is indicated. **g**, Mapping of the small RNA-seq data to CRISPR1 array. The data is representative of biological triplicates. A portion of the array is shown in Fig. 1b. R, repeat (light blue boxes); S, spacer (white boxes). **h**, SEC traces following affinity purification of His_6_-Cas6f co-expressed with different RNA variants: type I-F crRNA, RacrIF1, RacrIF1^GCmut^ or an empty vector (no RNA) control. The A_260_/A_280_ ratio is indicative of the presence of nucleic acids, and the RNA was isolated from these fractions and run in a denaturing gel (Fig. 1f).

To investigate the putative anti-CRISPR function of phage-encoded SRUs, we searched for previously identified candidates^4^ with similarity in sequence and secondary structure to the type I-F CRISPR repeats of *Pectobacterium* *atrosepticum* strain SCRI1043 (Extended Data Fig. 1a,b). Using these criteria we selected a type I-F SRU (PPOA865) that is encoded in an intergenic region of a *Thiocystis* *violascens* prophage (NC_018012.1; 4,752,620–4,811,169) (Fig. 1a,b and Extended Data Fig. 1a–c). Small RNA sequencing (RNA-seq) and 5′ rapid amplification of cDNA ends (RACE) of *P.* *atrosepticum* carrying this SRU and its flanking regions on a plasmid revealed it is expressed as a small non-coding RNA from its native promoter (Fig. 1c and Extended Data Fig. 1c–f). Next, *P.* *atrosepticum* expressing the I-F SRU was challenged with the virulent phage ΦTE when targeted by the endogenous type I-F CRISPR–Cas system^18^. Although ΦTE was efficiently restricted, expression of the I-F SRU gave the phage a strong replicative advantage (Fig. 1d). We then found that the inhibitory effect of the SRU was independent of the invading element, because SRU expression also protected a targeted plasmid during conjugation (Fig. 1e). Together, these results show that this small non-coding RNA has strong anti-CRISPR activity against the type I-F CRISPR–Cas immune response. We therefore refer to this SRU as RNA anti-CRISPR IF1 (RacrIF1).

## Cas6f binds to and processes RacrIF1

**Extended Data Fig. 2:**
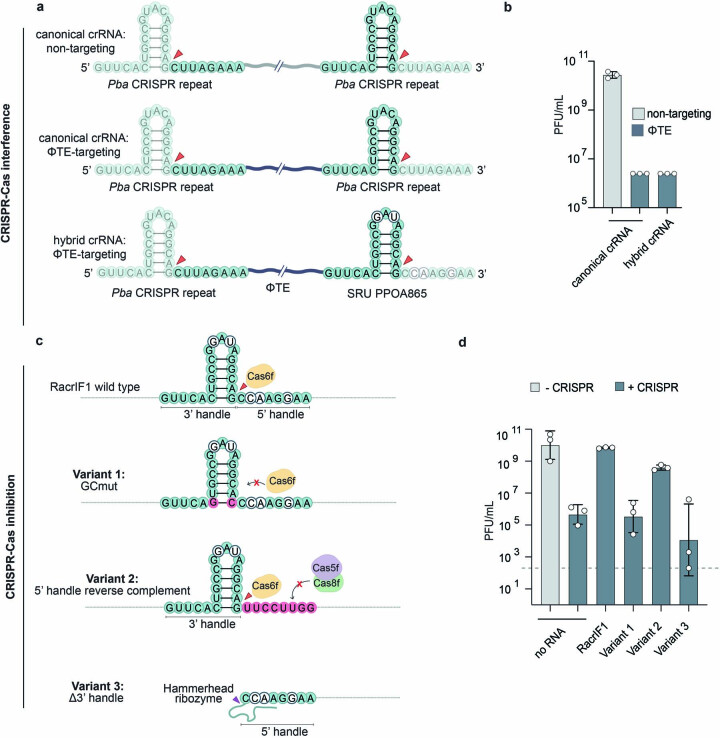
The 3′ stem-loop (upstream) region of RacrIF1 provides anti-CRISPR activity. **a**, Schematic of non-targeting and ΦTE-targeting crRNAs composed of different combinations of the RacrIF1 repeat and the *Pba* repeat to test targeting capacity. **b**, PFU/mL for ΦTE infecting *Pba* non-targeting (–CRISPR, grey) or targeting the phage (+CRISPR, blue), carrying either a targeting or non-targeting canonical *Pba* crRNA or a hybrid crRNA. **c**, Schematic of the RacrIF1 mutant variants generated for functional characterization. Variant 1, C6G/G20C mutation at the base of the stem in RacrIF1; variant 2, reverse complement mutation in the 5′ handle; variant 3, stem-loop deletion and introduction of a hammerhead ribozyme for Cas6f-independent processing. **d**, PFU/mL for ΦTE infecting *Pba* non-targeting (–CRISPR, grey) or targeting the phage (+CRISPR, blue), carrying either an empty vector control (no RNA), a plasmid encoding wild-type RacrIF1 or variants thereof expressed from the P_BAD_ promoter. The dotted line indicates the detection limit. Data points displayed on the detection limit are below the detection limit. Data in **b** and **d** represent biological replicates (n = 3) plotted as the mean ± SD. Source data

In type I-F CRISPR–Cas systems, the endoribonuclease Cas6f (also known as Csy4) binds with high affinity to the repeat stem–loop structures in pre-crRNAs, cleaving them at the base to produce mature crRNAs^19^ (Fig. 1b and Extended Data Fig. 1g). Cas6f remains bound to the cleaved product and the other Cas proteins assemble along the crRNA to form a functional surveillance complex termed type I-F CRISPR-associated complex for antiviral defence (Cascade, or Csy complex)^20–22^. Given the high sequence identity and predicted secondary-structure similarity between RacrIF1 and the *P.* *atrosepticum* type I-F CRISPR repeat (Fig. 1a,b and Extended Data Fig. 1a,b), we proposed that RacrIF1 interacts with Cas6f. In support of this idea, Cas6f co-expressed with either RacrIF1 or the crRNA control co-purified with RNA species of similar size and abundance, whereas no discrete nucleic acids were detected in the absence of RacrIF1 or crRNA (Fig. 1f and Extended Data Fig. 1h). Consistent with previous work^19^, the length of the co-purified RNAs corresponds to the size of the crRNA fragment protected by Cas6f after binding and processing; that is, about 16 nucleotides (nt) (Fig. 1f). Moreover, mutation of the C6 and G20 nucleotides at the base of the stem in RacrIF1 (RacrIF1^GCmut^; Fig. 1a), which is a motif crucial for Cas6f pre-crRNA binding and processing^23^, abrogated Cas6f binding and any inhibitory effect of RacrIF1 (Fig. 1d,f and Extended Data Fig. 1h). Small RNA-seq revealed the canonical Cas6f processing site of RacrIF1 at the 3′ end of the stem–loop (Fig. 1c and Extended Data Fig. 1d), supporting the specificity of the Cas6f–RacrIF1 interaction. Consistent with the RNA abundance profiles of the *P.* *atrosepticum* crRNAs (Fig. 1b and Extended Data Fig. 1g) and previous studies^24,25^, the 5′ OH end of the 5′ handle produced during Cas6f processing of RacrIF1 is more stable than the transcript containing the 5′ PPP end derived from transcription initiation arising from RNA decay by 5′ pyrophosphate removal^19,26,27^ (Fig. 1c and Extended Data Fig. 1d). In support of Cas6f-mediated processing of RacrIF1, the addition of one type I-F repeat and a spacer targeting ΦTE to the RacrIF1 repeat resulted in a functional crRNA (Extended Data Fig. 2a,b). Taken together, our data demonstrate that RacrIF1 specifically binds to, and is processed *in vivo* by, Cas6f in a crRNA-like fashion.

To investigate which regions of RacrIF1 are crucial for its inhibitory effect, we tested variants for their ability to inhibit CRISPR–Cas targeting of ΦTE (Extended Data Fig. 2c). Modifications that disrupt Cas6f binding and processing (that is, those in the stem–loop) resulted in the complete loss of RacrIF1 inhibition (Fig. 1d and Extended Data Fig. 2c,d). By contrast, modifications to the 8-nt 5′ handle of the processed product of RacrIF1, which would exclude potential interactions with Cas5f and Cas8f^28–30^, still enabled substantial CRISPR–Cas inhibition (Extended Data Fig. 2c,d). Consistent with this finding, a variant of RacrIF1 generated by ribozyme processing that contained only the 5′ handle was not inhibitory (Extended Data Fig. 2c,d). Taken together, these data demonstrate that the RacrIF1 product upstream of the Cas6f processing site primarily accounts for CRISPR–Cas inhibition.

## RacrIF1 prevents Cascade formation

**Fig. 2:**
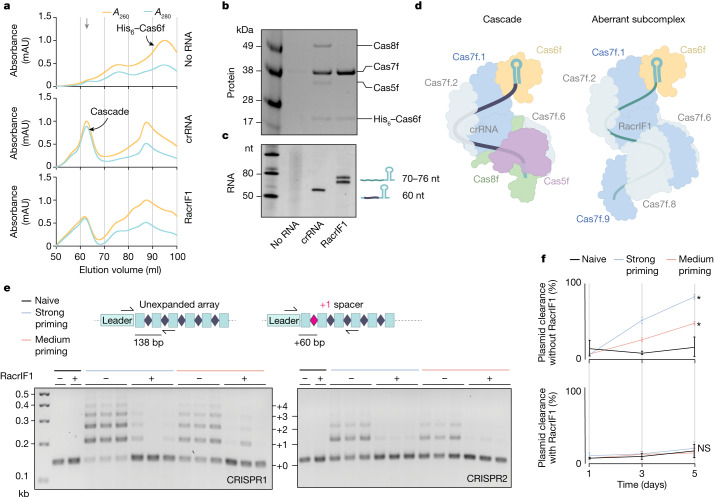
RacrIF1 prevents the formation of a canonical Cascade, inhibiting primed acquisition and plasmid clearance. **a**, SEC traces resulting from the co-expression of *cas8f*, *cas5f*, *cas7f* and *his*_*6*_*–cas6f* with no RNA (top), crRNA (middle) or RacrIF1 (bottom) from the P_BAD_ promoter in* E.* *coli*. The downward grey arrow indicates the fractions used in **b** and **c**. Graphs show absorbance (*A*) at wavelengths of 260 (orange) and 280 (blue) nm. mAU, milliabsorbance units. **b**, SDS–PAGE of protein fractions purified by SEC (selected fractions are indicated with a grey arrow in **a**). **c**, Denaturing urea PAGE of RNA isolated from protein fractions of the no RNA control, crRNA control and RacrIF1 sample (for gel source data, see Supplementary Fig. 1). **d**, Schematic of the type I-F Cascade complex and the predicted aberrant subcomplex formed around RacrIF1. **e**, CRISPR adaptation measured by expansion of the *P.* *atrosepticum* type I-F arrays (CRISPR1, left; CRISPR2, right) after 5 days of passaging cells that contain strong (blue) or medium (orange) priming-inducing plasmids, compared with a naive control (no matching protospacer, black). Cells contained a second plasmid expressing RacrIF1 (+) from the P_BAD_ promoter or an empty vector control (–). Data shown represent *n* = 3 biological replicates (for gel source data, see Supplementary Fig. 1). **f**, Percentage of cells (from **e**) that cleared the type I-F strong (blue) or medium (orange) priming-inducing plasmids compared with a naive control (no matching protospacer, black). *P.* *atrosepticum* strains contained a second plasmid expressing RacrIF1 from the P_BAD_ promoter (bottom) or an empty vector control (top). Flow cytometry was used to quantify the plasmid-encoded mCherry signal. Data shown represent *n* = 3 biological replicates plotted as mean ±s.d. Statistical significance was assessed using a two-way ANOVA test of primed samples compared with the naive control (*P ≤ 0.05). Source data

**Extended Data Fig. 3:**
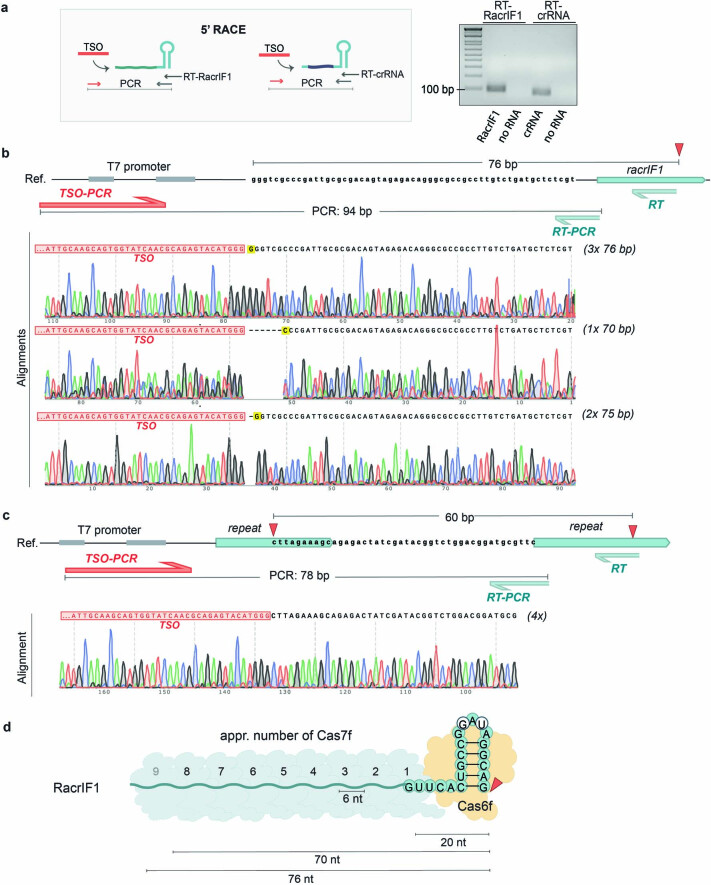
RacrIF1 5′ RACE analysis. **a**, Schematic of 5’ RACE analysis of the RNA products purified from the protein complexes in Fig. 2b,c with sequence specific reverse transcription (RT) primers for the RacrIF1 or the crRNA. The “no RNA” extracts from Fig. 2b,c served as negative controls. The resulting PCR products were visualized on gel (for gel source data, see Supplementary Fig. 1). **b**, Sanger sequencing results of 5′ RACE products retrieved from RacrIF1 subcloned on vector and mapped back to RacrIF1 expressed from T7 promoter used for protein purification. Six clones were sequenced and representatives of each product are shown. The expected size of the PCR and length of RacrIF1 from processing site to TSS are indicated. **c**, Sanger sequencing result of 5′ RACE products retrieved from crRNA subcloned on vector and mapped back to crRNA sequence expressed from T7 promoter used for protein purification. Four clones were sequenced and a representative is displayed. **d**, Schematic of RacrIF1 interacting with Cas6f and approximately eight to nine Cas7f subunits based on length of RacrIF1, where each Cas7f binds 6 nt^33,77^.

The type I-F Cascade is a complex of around 350 kDa composed of nine functionally necessary Cas proteins (one Cas6f, six Cas7f, one Cas5f and one Cas8f) and a 60-nt crRNA that makes direct contact with all the subunits^20^. We wanted to investigate whether RacrIF1 also interacts with additional Cas proteins and if it can support the formation of a complete Cascade complex. We therefore expressed in *Escherichia* *coli* the proteins of the type I-F *P.* *atrosepticum* Cascade with either RacrIF1 or a canonical type I-F crRNA, and purified the resulting complex by affinity purification of His_6_-Cas6f. Interestingly, we observed similar elution peaks during size-exclusion chromatography (SEC) in the presence of crRNA and RacrIF1 (Fig. 2a). However, SDS polyacrylamide gel electrophoresis (SDS–PAGE) revealed that although all the expected Cascade subunits were present with the crRNA (Fig. 2b), the expression with RacrIF1 led to the formation of an aberrant subcomplex. The subcomplex included Cas6f and Cas7f but lacked both Cas5f and Cas8f (Fig. 2b), which are required for target recognition^28,29^ and recruitment of the Cas2/Cas3 helicase–nuclease for interference^20,31^. The relative intensity of Cas7f was higher in the RacrIF1 complex, indicating that the ratio of Cas7f to Cas6f was higher than in the crRNA-containing complex. Consistent with this, RNA extraction revealed species of different sizes bound to the respective protein fractions. Whereas the crRNA control showed the expected size of 60 nt^32^, RacrIF1 was co-purified with two longer RNA species (Fig. 2c). 5′ RACE confirmed that the purified RNA corresponded to the RacrIF1 products upstream of the Cas6f processing site with lengths of approximately 70 nt and 76 nt (Extended Data Fig. 3a–c). Considering the RacrIF1 ribonucleoprotein-complex retention time on SEC, the increased Cas7f intensity and the length of purified RNA, we estimate that RacrIF1 supports a subcomplex comprising Cas6f and a long Cas7f filament of either eight or nine subunits oligomerized along the RNA^33^ (Fig. 2d and Extended Data Fig. 3d).

**Extended Data Fig. 4:**
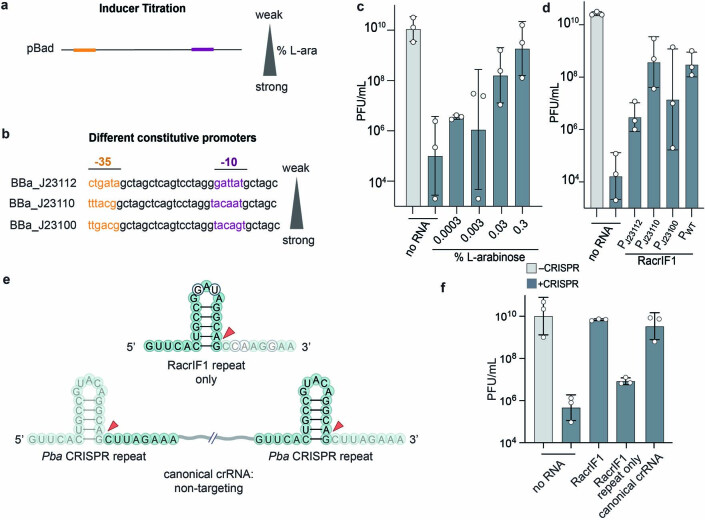
Dosage response of RacrIF1. **a**, Schematic of inducer-based titration of the P_BAD_ promoter with different concentrations of l-arabinose (L-ara). **b**, Overview of BioBrick constitutive promoters used to vary RacrIF1 expression. The −35 and −10 boxes are indicated. **c**, PFU/mL for ΦTE infecting *Pba* non-targeting (–CRISPR, grey) or targeting the phage (+CRISPR, blue), carrying either an empty vector control (no RNA) or a plasmid encoding the RacrIF1 expressed from the P_BAD_ promoter and induced at different L-ara concentrations. **d**, PFU/mL for ΦTE infecting *Pba* non-targeting (–CRISPR, grey) or targeting the phage (+CRISPR, blue), carrying either an empty vector control (no RNA), a plasmid encoding the RacrIF1 expressed from either its wild-type promoter or different BioBrick constitutive promoters. **e**, Schematic of RacrIF1 repeat-only and a canonical, non-targeting crRNA used for inhibition assays in **f**. **f**, PFU/mL for ΦTE infecting *Pba* non-targeting (–CRISPR, grey) or targeting the phage (+CRISPR, blue), carrying either an empty vector control (no RNA), a plasmid encoding the RacrIF1, the RacrIF1 repeat-only, or a canonical crRNA. Data in **c**, **d** and **f** represent biological replicates (n = 3) plotted as the mean ± SD. Source data

**Extended Data Fig. 5:**
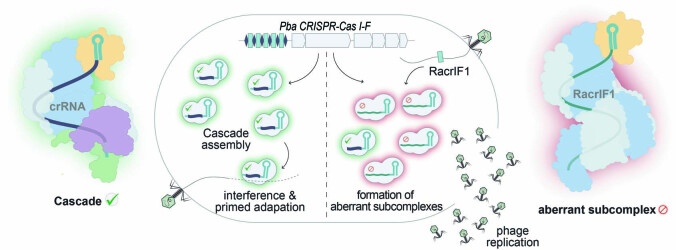
Schematic of proposed RacrIF1 mechanism. Canonical CRISPR–Cas interference (left). A functional type I-F Cascade is guided by the crRNA and clears phage infection. RacrIF1 inhibits the CRISPR–Cas response (right). RacrIF1 is expressed from the phage genome under a strong promoter and competes with host crRNAs for Cas6f and Cas7f subunits. The resulting aberrant subcomplex is non-functional for interference and outnumbers the functional interference complex. Ultimately, the infecting phage can replicate and spread.

We reasoned that RacrIF1 acts as a competitive inhibitor of type I-F Cascade by forming an aberrant subcomplex. Consistent with this idea, titrating RacrIF1 expression (by varying an inducible promoter or using a series of promoters with different strengths) revealed dose-dependent CRISPR–Cas inhibition (Extended Data Fig. 4a–d). Importantly, the native RacrIF1 is highly expressed from its wild-type promoter, because it provides a level of CRISPR–Cas inhibition similar to that of a strong constitutive promoter (Extended Data Fig. 4b,d). Consistent with a model in which RacrIF1 competes with phage-targeting crRNAs for Cas proteins, the expression of either RacrIF1 or a non-targeting crRNA reduced CRISPR–Cas interference and allowed phage infection (Extended Data Fig. 4e,f). Taken together, these data indicate that RacrIF1 promotes the formation of an aberrant subcomplex, sequestering Cas proteins away from endogenous targeting crRNAs, thereby protecting the phage from CRISPR–Cas immunity (Extended Data Fig. 5).

## RacrIF1 inhibits primed adaptation

**Extended Data Fig. 6:**
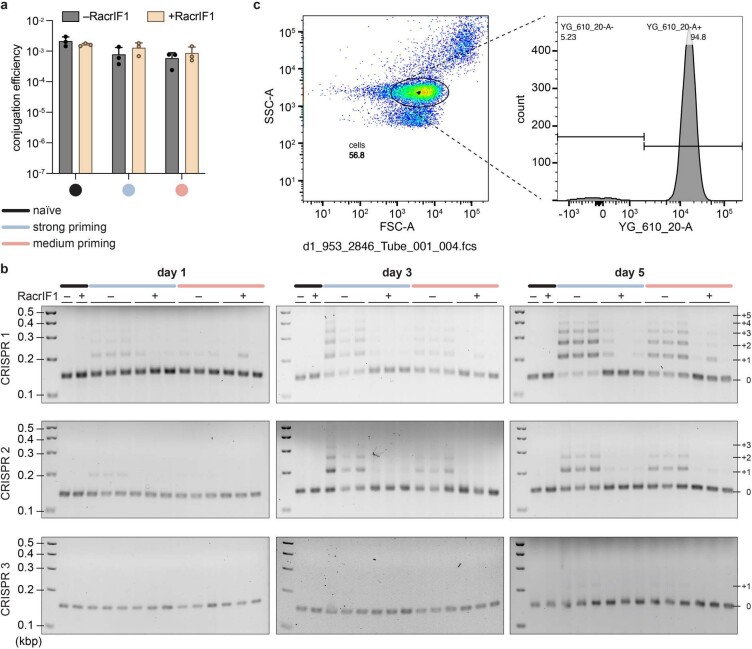
RacrIF1 inhibits primed adaptation. **a**, Conjugation efficiency assay of a type I-F strong (AG PAM variant, blue) or a medium (GT PAM variant, orange) priming-inducing plasmids into *Pba* compared with a naïve control (no matching protospacer, black). Cells were carrying either a plasmid expressing RacrIF1 (+RacrIF1, yellow) or an empty vector control (–RacrIF1, gray). Data shown represent biological replicates (n = 3) plotted as the mean ± SD. **b**, CRISPR adaptation measured by expansion of the *Pba* type I-F arrays (CRISPR1, CRISPR2 and CRISPR3) after 1, 3 and 5 days of passaging strong (AG PAM variant, blue) or medium (GT PAM variant, orange) priming-inducing plasmids compared with a naïve control (no matching protospacer, black). Cells contained a second plasmid expressing RacrIF1 (+RacrIF1) or an empty vector control (–RacrIF1). Data shown represent biological replicates (n = 3) (for gel source data, see Supplementary Fig. 1). **c**, Flow cytometry gating strategy adopted for plasmid clearance assay in Fig. 2f.

CRISPR–Cas immunity hinges on the ability to acquire new spacers from invaders. Although spacer acquisition from an unknown invader (naive adaptation) is a rare event, it is greatly accelerated in type I systems by crRNAs that perfectly or imperfectly (for PAM or protospacer variants) match a target. This process is called primed adaptation and requires cooperation between the adaptation and interference machineries^34^. Given that RacrIF1 competes for Cas proteins with host crRNAs through the formation of an aberrant subcomplex, we speculated that it would also compromise primed acquisition. To explore this, we performed a priming assay by introducing plasmids containing protospacers with variant PAMs into *P.* *atrosepticum.* These plasmids stimulate acquisition owing to imperfect targeting^35^ from chromosomal spacer S1 (Extended Data Fig. 6a). We then monitored the effect of RacrIF1 on the expansion of the native *P.* *atrosepticum* CRISPR arrays. We observed high spacer-acquisition rates in the absence of RacrIF1, whereas minimal expansion was detected in populations that express RacrIF1 (Fig. 2e and Extended Data Fig. 6b). Because the acquisition of spacers leads to interference and loss of the priming-inducing plasmids^36^, we quantified plasmid clearance by measuring mCherry expression from the plasmids (Extended Data Fig. 6c). As expected, rapid plasmid clearance was observed in the absence of RacrIF1, because the acquisition of spacers through priming led to plasmid interference (Fig. 2f). By contrast, the priming-inducing plasmids were stably maintained in populations that express RacrIF1, in a similar way to the naive plasmids, indicating that the differences in plasmid clearance were strictly dependent on RacrIF1 (Fig. 2f). In conclusion, these data demonstrate that RacrIF1 inhibits both primed adaptation and the resulting CRISPR–Cas targeting, inducing an immunosuppressed state in the host that reduces its capacity to adapt to infection.

## Racr candidates are diverse and widespread

**Fig. 3:**
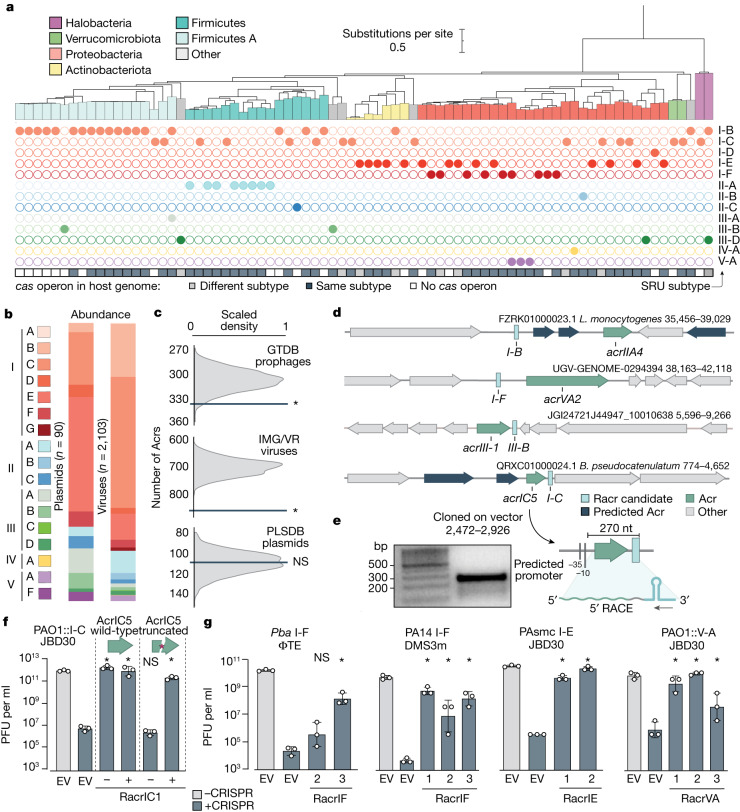
Racrs are widespread across MGEs and are encoded adjacent to *acr* genes. **a**, Phylogenetic tree of bacterial hosts that contain SRU-carrying proviruses (classified according to the GTDB). The Racr subtype is indicated by coloured circles. CRISPR–Cas in the host chromosome is indicated as the same subtype (dark grey), a different subtype (light grey) or absent system (white). **b**, Relative abundance of SRUs identified in viruses (IMG/VR3 viruses and GTDB proviruses) and plasmids (PLSDB). **c**, Number of putative *acr* genes within 1 kb of a *racr* candidate. The dark grey line depicts the observed number, whereas the density plot represents 1,000 random permutations of the Racr candidate positions. Statistical significance was assessed through permutation test, one-tailed; *P ≤ 0.05. **d**, Genomic organization of colocalizing *racr* candidates and *acr* genes. Bacteria mentioned are *Listeria monocytogenes* and *B.* *pseudocatenulatum*. **e**, 5′ RACE analysis of the type I-C *acr*–*racr* locus cloned in an expression vector with its wild-type promoter (for gel source data, see Supplementary Fig. 1). **f**, PFU per ml for JBD30 infecting wild-type PAO1 (–CRISPR, grey) or PAO1::IC (+CRISPR, blue) having an empty vector control (EV), the full *acr*–*racr* locus or the *acr*–*racr* locus with a truncated *acrIC5* with (+) and without (–) RacrIC1 expressed from the P_BAD_ promoter. **g**, PFU per ml for phages ΦTE, DMS3m and JBD30 against different CRISPR–Cas systems: subtypes I-F (*P.* *atrosepticum* (Pba) and PA14), I-E (PAsmc) and V-A (*Moraxella bovoculi*) in PAO1 (PAO1::V-A). Serial dilutions of each phage were spotted on their cognate host non-targeting (–CRISPR, grey) or targeting (+CRISPR, blue) the phage. Cells contained a plasmid expressing different Racr candidates from the P_BAD_ promoter or an empty vector (EV) control. Data in **f** and **g** represent *n* = 3 biological replicates plotted as the mean ± s.d. Statistical significance was assessed using one-way ANOVA test of +Racr samples compared with the EV +CRISPR control (*P ≤ 0.05). Source data

**Extended Data Fig. 7:**
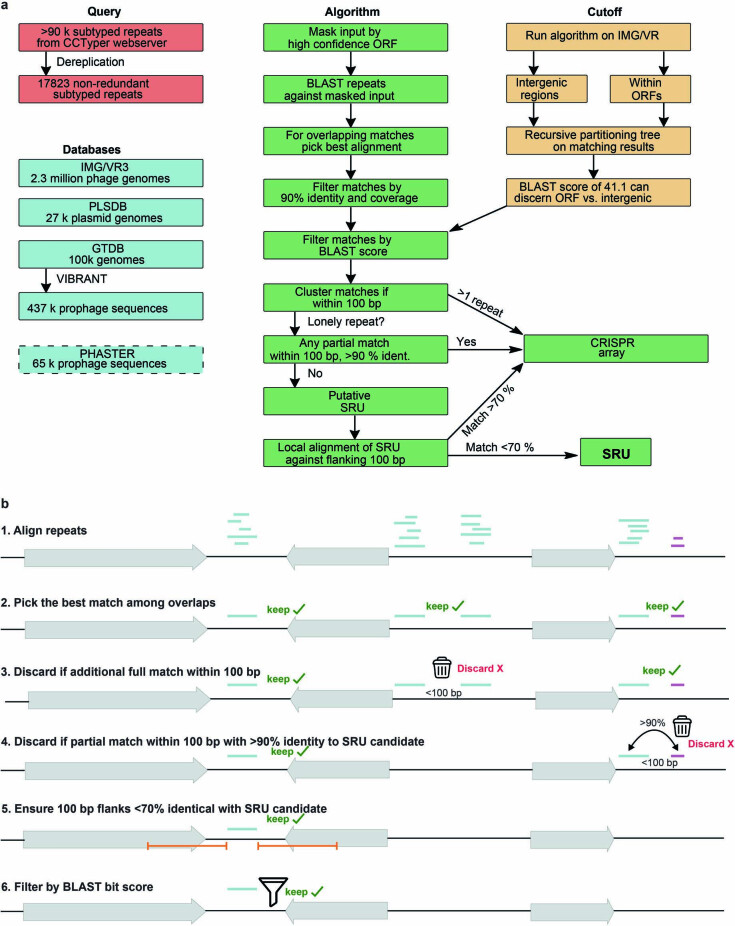
Bioinformatic identification of SRUs. **a**, Flowchart of the SRUfinder^40^ pipeline displaying the bioinformatic pipeline used for finding SRU candidates in DNA sequences. **b**, Schematic of the decision process on SRU sequence identification on sequence level whether a SRU was kept or discarded in six consecutive steps.

**Extended Data Fig. 8:**
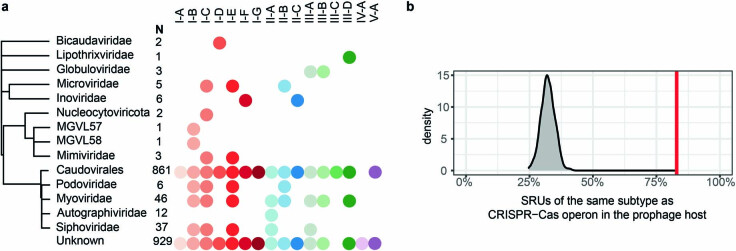
SRUs are encoded on different types of viruses and infect hosts that carry the same CRISPR–Cas type. **a**, Phage taxonomy dendrogram and presence of SRUs. N refers to the total number of virus genomes with any SRU. **b**, Non-random association between the CRISPR–Cas subtype prediction of prophage SRUs and the *cas* operon(s) found in the host chromosomes carrying the prophages. 83% of SRUs match the subtype of the corresponding *cas* operon(s) encoded in the host. When the SRU subtype is randomly assigned (1000 permutations), the mean association is 32.3% with a standard deviation of 2.7%, as shown in the density plot. The analysis only includes data of SRUs retrieved from the GTBD database for which the host also encodes CRISPR–Cas (n = 188).

To gain a deeper understanding of the diversity and distribution of SRUs and, therefore, of potential Racr candidates, we performed an extensive search across MGE sequence datasets, including prophage sequences in the Genome Taxonomy Database (GTDB)^37^, plasmids in the PLSDB^38^ and viral metagenomic data in the Integrated Microbial Genomes Viral Resources (IMG/VR3) database^39^. Our dedicated algorithm, SRUFinder^40^, uses an updated repeat database of 17,823 non-redundant sequences as the query, including subtype representatives of all known CRISPR–Cas systems (classes 1 and 2). We focused our search on intergenic regions and identified hits that matched known repeats but that were not genetically associated with other (partial) repeats (Extended Data Fig. 7). This analysis revealed that MGEs that encode SRUs include different types of virus (Extended Data Fig. 8a) that infect a diverse range of hosts, including bacterial and archaeal taxa (Fig. 3a). Notably, around 90% of prophages and viruses that encode SRUs infect hosts that have CRISPR–Cas systems (Fig. 3a). Of these SRUs, 83% matched the predicted subtype of the corresponding host CRISPR–Cas system (Extended Data Fig. 8b and Supplementary Data 1), indicating that they may interact with host CRISPR–Cas as Racrs. Across all databases, we identified 2,103 SRUs on viral sequences and 90 SRUs on plasmids, including subtype representatives of all CRISPR–Cas types except the uncommon type VI (Fig. 3b and Supplementary Data 2).

## Candidate *racr* genes cluster with *acr* genes

**Extended Data Fig. 9:**
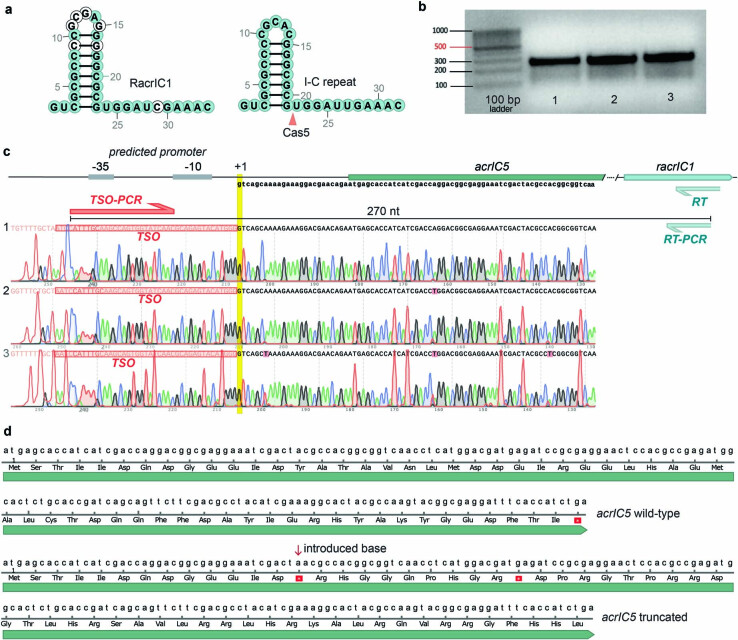
RacrIC1 and AcrIC5 are encoded in the same bicistronic RNA and both inhibit type I-C CRISPR–Cas. **a**, Secondary structure of RacrIC1 and a type I-C CRISPR repeat. In type I-C, Cas5 is responsible for crRNA processing^78,79^. **b**, 5′ RACE analysis of the identified *acr-racr* locus in *B. pseudocatenulatum* cloned in an expression vector under wild-type promoter expression in triplicates (for gel source data, see Supplementary Fig. 1). **c**, Sanger sequencing results of PCR products acquired through 5′ RACE and confirmation of exact TSS. Primers used are indicated. **d**, Zoom in on the AcrIC5 sequence. Truncation of AcrIC5 introduced by single nucleotide insertion causing preliminary stop codons in the sequence.

Previously identified *acr* genes are frequently co-located in MGEs with genes of similar function (such as other *acr* genes) in so-called anti-CRISPR loci or anti-defence gene clusters^2,5^. We therefore hypothesised that *acr* genes will frequently be found next to *racr* genes. In support of this idea, we observed a significant enrichment of predicted *acr* genes within 1 kb of identified *racr* gene candidates in viral sequences (Fig. 3c). There was no association for plasmids, possibly because there are insufficient data for plasmid-encoded SRUs (*n* = 90; Fig. 3b). Furthermore, several Racr candidates are encoded adjacent to homologues of functionally validated *acr* genes (Fig. 3d). Among these, we found a type I-C Racr candidate encoded immediately downstream (11 nt) of a prophage-encoded AcrIC5 homologue in *Bifidobacterium pseudocatenulatum* strain AF18-2AC (Fig. 3d and Extended Data Fig. 9a). This genetic architecture led us to speculate that the putative *racr* and *acrIC5* genes are in the same operon, as described for several anti-CRISPR loci^5,41^. Indeed, 5′ RACE of this locus under wild-type promoter expression in *Pseudomonas aeruginosa* PAO1 confirmed that the two genes are expressed as a bicistronic RNA, indicating their functional relatedness (Fig. 3e and Extended Data Fig. 9b,c). Expression of the putative *acr*–*racr* locus in PAO1 carrying a type I-C CRISPR–Cas system (hereafter PAO1::IC) restored the infectivity of a targeted phage (JBD30), confirming the CRISPR-inhibitory role of this operon (Fig. 3f). To untangle the contributions of the Acr protein and the Racr candidate, we co-expressed the wild-type and a truncated version of AcrIC5 with and without the predicted I-C *racr* downstream (Extended Data Fig. 9d). Both the wild-type AcrIC5 and the Racr candidate (RacrIC1) individually provided the phage with a strong replicative advantage in the presence of type I-C targeting (Fig. 3f). Our results reveal the frequent co-localization of *racr* and *acr* genes across viral genomes, further supporting their pervasive involvement in CRISPR–Cas inhibition.

## Racrs inhibit distinct CRISPR–Cas classes

**Extended Data Fig. 10:**
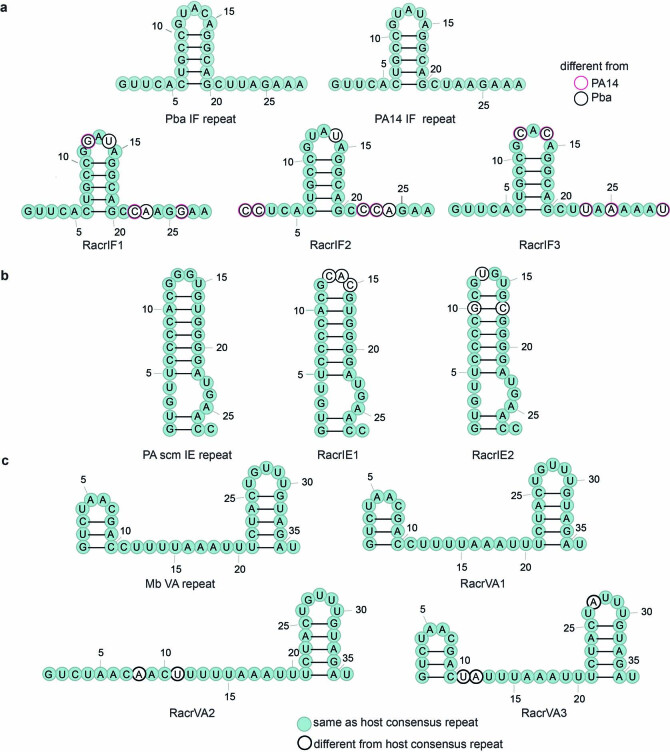
Secondary structure of experimentally verified Racrs and related host CRISPR repeats. **a**, *Pba* and PA14 I-F consensus repeats compared to RacrIF1, -IF2, and -IF3. **b**, PAscm I-E consensus repeat compared to RacrIE1 and -IE2. **c**, Mb V-A consensus repeat compared to RacrVA1, -VA2, and -VA3.

We then investigated whether other predicted Racrs exhibit anti-CRISPR activity. We selected candidates with high sequence identity and predicted RNA secondary-structure similarity to the CRISPR repeats of multiple CRISPR–Cas subtypes (Extended Data Fig. 10a–c and Supplementary Table 1). Indeed, expression of additional I-F Racr candidates allowed phage ΦTE to overcome CRISPR–Cas immunity in *P.* *atrosepticum* (RacrIF2 and RacrIF3) (Fig. 3g). A similar inhibitory effect was observed for RacrIF1, RacrIF2 and RacrIF3 against the endogenous *P.* *aeruginosa* PA14 type I-F system, reflecting a broad host range activity (Fig. 3g). Furthermore, two I-E Racr candidates inhibited the *P.* *aeruginosa* SCM4386 (PAscm) type I-E system (RacrIE1 and RacrIE2) (Fig. 3g), and three V-A Racr candidates  (RacrVA1, RacrVA2 and RacrVA3) inhibited the *Moraxella* *bovoculi* type V-A system reconstituted in PAO1 (PAO1::V-A) (Fig. 3g). Taken together, our data demonstrate that many MGEs encode Racrs that inhibit CRISPR–Cas systems found across diverse taxa, including subtype representatives of the two CRISPR–Cas classes.

## Discussion

Evading host antiviral defences is an important part of infection, and many viruses use Acr proteins to block CRISPR–Cas immune functions^2^. Here we describe a mechanism widely used by MGEs to suppress CRISPR–Cas immunity through the use of SRUs as small non-coding RNA anti-CRISPRs or Racrs. SRUs had been previously identified bioinformatically in viral sequences, but their biological functions remained untested^4^. We show that a prophage-encoded Racr mimics cognate I-F CRISPR repeats and suppresses CRISPR–Cas immunity. Biochemical characterization showed that RacrIF1 is bound to, and processed by, the host endoribonuclease Cas6f, and that the product supports the formation of a Cas7f filament. However, the functionally essential proteins Cas5f and Cas8f are not present in the resulting subcomplex owing to the lack of a 5′ handle on the RNA component necessary for Cas protein recruitment^20,33^. By competing with endogenous crRNAs for Cas components, RacrIF1 prevents the formation of a functional Cascade, thereby inhibiting both CRISPR interference and primed adaptation (Extended Data Fig. 5). The consequences of this phenomenon are probably compounded over evolutionary timescales by impairing the ability of the host cell to respond to infection and promoting the retention of genetic invaders, even if they have previously been encountered.

We identified Racr candidates against almost all known CRISPR–Cas types in viruses and plasmids that infect diverse prokaryotic taxa, highlighting the independent evolution of CRISPR-repeat mimicry across MGEs. Functional testing of these putative Racrs against diverse CRISPR–Cas subtypes (I-C, I-E, I-F and V-A) confirmed their inhibitory functions. These results, together with the observed genetic co-location of bioinformatically predicted *racr* and *acr* genes (a strong indicator of anti-CRISPR function^5^), suggest the widespread use of this immune-evasion strategy. Their frequent co-location may further suggest the existence of cooperative interactions between Racrs and Acrs. It cannot be ruled out that some predicted Racrs have functions other than immune suppression. Indeed, other CRISPR-repeat-like sequences can mediate viral integration into CRISPR arrays^17^ or Cas-dependent transcriptional repression^13,42,43^.

Molecular mimicry of host defence components is not restricted to CRISPR–Cas and is a common strategy widely adopted by viruses to exploit and subvert host processes during infection. For example, some phages mimic antitoxin non-coding RNAs^18^ or host methyltransferases^44^ to ensure viral replication despite the presence of anti-phage defences. Furthermore, some viruses that infect humans co-opt tumour suppressors^45^, homologues of chemokine receptors^46^ or inhibitors of the complement system^47^. Such examples of convergent evolution highlight the double-edged nature of immune systems, the components of which have become practical targets for molecular mimicry and exaptation in the host–pathogen evolutionary arms race.

The past decade has seen a remarkable expansion of CRISPR–Cas biotechnologies and an increased demand to modulate their activities. Although the discovery of Acr proteins has led to tools for regulating and increasing the precision of CRISPR–Cas applications^6^, our work contributes to the continued exploration of harnessing nucleic-acid-based inhibitors for this purpose^48,49^. The discovery of new Acr proteins can be complex and time consuming, but the similarity of Racrs to known CRISPR repeats simplifies their identification and has great potential in enabling rational design strategies. We anticipate that further investigation of the properties of Racrs will not only provide a better understanding of phage–bacterial interactions, but will also create promising opportunities for the development of future molecular biology tools.

## Methods

### Bacterial strains and growth conditions

The bacterial strains used in this study are listed in Supplementary Table 2. Unless otherwise noted, the *P.* *atrosepticum*, *P.* *aeruginosa* (PA14, PAscm and PAO1) and *E.* *coli* strains were routinely grown *at* 25 °C, 30 °C and 37 °C, respectively, in lysogeny broth (LB) shaken at 180 rpm or on LB–agar (LBA) plates containing 1.5% (w/v) agar. When applicable, antibiotics and supplements were added at the following concentrations: ampicillin, 100 µg ml^−1^; chloramphenicol (Cm), 25 µg  ml^−1^; kanamycin, 50 µg  ml^−1^; gentamicin, 50 µg  ml^−1^ for *P.* *aeruginosa* or 15 µg  ml^−1^ for *E.* *coli*; tetracycline (Tc), 5 µg  ml^−1^; 5-aminolevulinic acid (ALA), 50 µg  ml^−1^; isopropyl β-D-1-thiogalactopyranoside (IPTG), 100 µM for *P.* *atrosepticum* or 1 mM for PAO1; l-arabinose (Ara), 0.3% (w/v). Bacterial growth was measured as the optical density at 600 nm (OD_600_) using a Jenway 6300 Spectrophotometer.

### Phage purification and titration

The phages used in this study are listed in Supplementary Table 2. In brief, 2 ml of overnight host culture was inoculated into 50 ml LB in a 250 ml flask and incubated for 30 min. Then 100 µl of phage lysate was added to the culture and incubated overnight. A centrifugation step was done (3,220*g* for 20 min at 4 °C) to separate the virions from the cell debris. The supernatant was placed in a sterile universal container for storage and a few drops of NaCO_3_-saturated chloroform were added before thoroughly vortexing the mixture to lyse any remaining cells. Finally, the phage titre was determined by pipetting 20-μl drops of serial dilutions of the phage stock in phage buffer (10 mM Tris-HCl, pH 7.4, 10 mM MgSO_4_, 0.01% (w/v) gelatin) onto an LBA overlay (0.35% w/v) seeded with 100 μl host overnight culture. Plaques were counted after incubation overnight, with the phage titre represented as PFU per ml. *Pseudomonas* phages DMS3m and JBD30 were propagated on PA14 ΔCRISPR, wild-type PAO1 or PAsmc Δ*cas3*. *Pectobacterium* phage ΦTE was propagated on wild-type *P.* *atrosepticum*. *Pseudomonas* phages were stored at 4 °C in SM buffer (50 mM Tris-HCl, pH 7.5, 100 mM NaCl, 8 mM Mg_2_SO_4_) over chloroform. *Pectobacterium* phage ΦTE was stored at 4 °C in phage buffer over chloroform.

### DNA isolation and manipulation

The oligonucleotides used in this study are listed in Supplementary Table 3. The polymerases, restriction enzymes, Gibson Assembly mix, USER enzyme and T4 ligase were obtained from New England Biolabs or Thermo Fisher Scientific. DNA from PCRs and agarose gels was purified using the Illustra GFX PCR DNA and Gel Band Purification Kit (GE Healthcare) or QIAEX II Gel Extraction Kit (Qiagen). Restriction digests, ligations and *E*. *coli* transformations were done using standard techniques. Plasmid DNA was extracted from overnight cultures using the Zyppy Plasmid Miniprep Kit (Zymo Research) or QIAprep Spin Miniprep Kit (Qiagen) and confirmed by DNA sequencing. Plasmids and their construction details are listed in Supplementary Table 4. Plasmids were introduced into *P.* *atrosepticum* and *P.* *aeruginosa* strains by electroporation using standard techniques.

### Selection and cloning of Racr candidates

Candidate Racrs were chosen on the basis of their similarity of sequence and secondary RNA structure to the relevant CRISPR repeats in the model system (MAFFT alignments, FastTree approximately maximum-likelihood phylogenetic trees; Extended Data Figs. 9a and 10a–c) and the presence of a promoter sequence within 250 bp upstream of the Racr candidate (promoter prediction using Bprom and manual curation). The Racr candidates were synthesized as gene fragments including flanking regions (Twist Biosciences) under the control of either the predicted wild-type promoter or P_BAD_ (Ara-inducible) from the predicted transcription start site (TSS). RacrIF1 variants were cloned through PCR with mismatched primers and overlap PCR. For variant 3, a hammerhead ribozyme was introduced for Cas6f-independent processing^50,51^. RacrIF1 was then cloned downstream of BioBrick constitutive promoters of different strength (BBa_J23112, BBa_J23110 and BBa_J23100) to evaluate dose responsiveness. Detailed information on the candidate Racrs is listed in Supplementary Table 1.

### Expression of Racr candidates and related constructs

For the experiments presented in Fig. 1d and Extended Data Figs. 2b,d and 4c,d,f, RacrIF1 and its variants, canonical and hybrid crRNAs and the isolated RacrIF1 repeat, were expressed in *P.* *atrosepticum* from a plasmid with a p15A origin of replication (copy number of around 10) either under the control of its wild-type promoter (NC_018012.1: 4,787,341–4,787,695 bp) or with the predicted TSS downstream of the P_BAD_ promoter (NC_018012.1: 4,787,535–4,787,695 bp) (Extended Data Fig. 1f). For the titration displayed in Extended Data Fig. 4c,d, RacrIF1 (NC_018012.1: 4,787,535–4,787,695 bp) was expressed from the P_BAD_ promoter under different Ara concentrations or the BioBrick constitutive promoters. For the experiments shown in Fig. 3f,g, Racr candidates tested in PAO1 or PA14 were expressed from the *Escherichia–Pseudomonas* (ColE1-pRO1600) shuttle vector pHERD30T with their predicted TSS downstream of the P_BAD_ promoter. RacrIF1 (experiment in Extended Data Fig. 1e) and RacrIC1 (experiment in Fig. 3e and Extended Data Fig. 9b) were cloned with the predicted wild-type promoter for the 5′ RACE assay. In *Pseudomonas* strains, pHERD30T replicates from the *P.* *aeruginosa* plasmid pRO1600 oriV and replication protein (copy number of around 13)^52,53^.

### Phage-resistance assay

Triplicate cultures of hosts carrying either a phage-targeting spacer (+CRISPR) or a non-targeting control (–CRISPR), and containing a plasmid expressing a candidate Racr or an empty-vector control (EV), were grown overnight in 5 ml LB supplemented with the appropriate antibiotics and inducers. For *P.* *atrosepticum*, a soft LBA overlay (0.35% w/v) containing 100 μl of the overnight cultures was poured onto an LBA plate supplemented with the corresponding antibiotics and inducers. For PA14, PAsmc and PAO1, a soft LBA overlay (0.5% w/v) containing 150 μl of the overnight cultures and supplemented with 10 mM MgSO_4_ was poured onto an LBA plate supplemented with 10 mM MgSO_4_ and the corresponding antibiotics and inducers. Phage titres were determined by pipetting 2.5 μl (or 5 μl for ΦTE) drops of serial dilutions of phage stock (approximately 10^10^ PFU per ml) in phage buffer onto the agar overlay and plates were incubated overnight. Plaques were counted after incubation overnight, with the phage titre represented as PFU per ml. When plaques were too small to count, one plaque was counted in the first dilution in which no plaques were visible. Type I-F Racr candidates were tested in *P.* *atrosepticum* PCF610 carrying the ΦTE targeting plasmid pPF1423 (for assays in Figs. 1d, 3g and Extended Data Figs. 2b and 4d) and *P.* *atrosepticum* PCF188 (for assays in Extended Data Figs. 2d and 4c,f) with the phage ΦTE, and *P.* *aeruginosa* PA14 with the phage DMS3m. Type I-E Racr candidates were tested in PAsmc, type V-A Racrs in PAO1::MbCpf1::crRNA24 (PAO1::V-A) and type I-C Racrs in PAO1 tagged with a I-C CRISPR–Cas system (PAO1::I-C), all of which were infected with the phage JBD30. The respective non-targeting (–CRISPR) control strains were *P.* *atrosepticum* PCF610 with the non-targeting plasmid pPF975 or wild-type *P.* *atrosepticum*, PAscm Δ*cas3* and wild-type PAO1.

### Conjugation-efficiency assay

For the experiment shown in Fig. 1e, conjugation efficiency was assessed in a similar manner to that described previously^54^. *E.* *coli* ST18 was the donor for the conjugation of the untargeted control (–CRISPR, pPF953) and type I-F (+CRISPR, pPF954) targeted plasmids. Plasmid pPF954 contains a protospacer targeted by spacer 1 from CRISPR1 (type I-F) and the canonical GG PAM. Recipients were wild-type *P.* *atrosepticum* that have either a plasmid expressing RacrIF1 from the P_BAD_ promoter (+RacrIF1, pPF2846) or an empty-vector control (–Racr1F1, pPF781). Strains were grown overnight in triplicate in 5 ml LB supplemented with Cm and Ara for recipients, or 5 ml LB supplemented with Tc and ALA for donor strains. One ml of overnight culture was pelleted and washed twice with LB supplemented with ALA to remove the antibiotics. Pellets were resuspended in 0.5 ml LB supplemented with ALA and Ara, and the OD_600_ was adjusted to 1. Donors and recipients were mixed in a 1:1 ratio, and 10 μl was spotted on LBA supplemented with ALA and Ara, and incubated at 25 °C for 24 h. Next, the mating spots were scraped with a sterile loop and resuspended in 0.5 ml PBS, and dilution series were plated either onto LB supplemented with Cm and Ara for recipient counts or with the addition of Tc for selection of transconjugant counts. Conjugation efficiency was calculated as the ratio of transconjugants per recipient cells.

### Co-expression and purification of Cas6f and RNA

For co-expression and purification of Cas6f and RNA variants, plasmids pPF2644 (His_6_–Cas6f and type I-F crRNA repeat–spacer–repeat), pPF2868 (His_6_–Cas6f and RacrIF1), pPF2869 (His_6_–Cas6f and RacrIF1^GCmut^) and pPF2640 (His_6_-Cas6f alone) were transformed into *E.* *coli* LOBSTR cells. Overnight cultures were used to inoculate 500 ml LB plus kanamycin in a 2 l baffled flask and incubated at 37 °C and 180 rpm to an OD_600_ of 0.2–0.3, followed by incubation at 18 °C and 180 rpm to an OD_600_ of 0.6. Expression was induced with 1 mM IPTG, and proteins were expressed for 20 h at 18 °C and 180 rpm. Cells were collected at 10,000*g* for 10 min at 4 °C, and the pellet was resuspended in 10 ml g^−1^ (wet-cell mass) lysis buffer (50 mM HEPES-NaOH, pH 7.5, 300 mM KCl, 5% (v/v) glycerol, 1 mM dithiothreitol (DTT) and 10 mM imidazole) supplemented with 0.02 mg ml^−1^ DNase I, one tablet cOmplete EDTA-free protease inhibitor (Roche), 0.67 mg  ml^−1^ lysozyme and 0.1 mM phenylmethylsulfonyl fluoride. Cells were lysed by ultrasonication and the lysate was clarified by centrifugation at 15,000*g* for 15 min at 4 °C. The cleared lysate was affinity purified using a 1 ml HisTrap™ FF (Cytiva) column equilibrated in lysis buffer and eluted using a gradient against elution buffer (lysis buffer containing 500 mM imidazole). Elution fractions were pooled and concentrated using a 10 kDa Nominal Molecular Weight Limit Amicon Ultra-4 Centrifugal Filter Unit (Amicon) and loaded onto a Superdex 75 Increase 10/300 GL (GE Healthcare) column equilibrated in SEC buffer (20 mM HEPES-NaOH, pH 7.5, 100 mM KCl, 5% (v/v) glycerol and 1 mM DTT). Protein concentrations were determined using a NanoDrop One Spectrophotometer (Thermo Fisher) and a Qubit Protein Assay Kit (Invitrogen). Aliquots of protein were stored at −80 °C. Protein samples were separated on an SDS–PAGE gel (Bolt 4 to 12%, Bis-Tris, 1,0 mm (Invitrogen)) and stained with Coomassie blue.

### Expression and purification of type I-F Cascade

For expression and purification of the type I-F Cascade shown in Fig. 2b, plasmids pPF1635 (Cas8f–Cas5f–Cas7f) and pPF2644 (His_6_–Cas6f and type I-F crRNA repeat–spacer–repeat) or pPF2868 (His_6_–Cas6f and RacrIF1) were co-transformed into *E.* *coli* LOBSTR cells. Protein was expressed and purified as described above with the following modifications: lysis buffer contained 15 mM imidazole, elution fractions were pooled and concentrated using a 30 kDa Nominal Molecular Weight Limit Amicon Ultra-4 Centrifugal Filter Unit (Amicon) and concentrated samples were loaded onto a HiLoad 16/600 Superdex 200 pg (GE Healthcare) column equilibrated in SEC buffer.

### RNA isolation from protein fractions

For the experiment shown in Fig. 2c, the different RNA variants were isolated from the purified His_6_–Cas6f or type I-F complex by phenol–chloroform extraction, ethanol precipitation and resolved on a denaturing gel containing 15% (v/v) 19:1 polyacrylamide, 7 M urea and 0.5× TBE (45 mM Tris, 45 mM Boric acid, pH 8.3, 1 mM EDTA) (Novex). The gel was stained with SYBR gold (Invitrogen) and RNA was shown using the Odyssey Fc imaging system (LICOR). For samples with purified His_6_–Cas6f only, the amount of protein was normalized before RNA isolation.

### Small RNA extraction and sequencing

For the experiments shown in Fig. 1b,c and Extended Data Fig. 1d,g, triplicate cultures of wild-type *P.* *atrosepticum* that have either a plasmid expressing RacrIF1 from its wild-type promoter (+RacrIF1, pPF2845) or an empty-vector control (–RacrIF1, pPF781) were grown overnight in 5 ml LB supplemented with Cm. The overnight cultures were subcultured into 25 ml LB supplemented with Cm in 250-ml flasks from a starting OD_600_ of 0.05 and incubated for 15 h up to stationary phase while monitoring culture growth (OD_600_). Next, 1 ml (in triplicate) of each culture was centrifuged for 1 min at 13,000*g*. The supernatant was discarded and the pellet was resuspended in 1 ml RNAlater Stabilization Solution (Invitrogen) and stored at −20 °C. The small RNA fraction (less than 200 nt) was extracted using the mirVana miRNA Isolation Kit according to the manufacturer’s instructions. Residual genomic DNA was removed by treatment with TurboDNase (Thermo Fisher) according to the manufacturer’s instructions, and the absence of gDNA was confirmed by PCR. RNA purity, integrity and concentration were determined using a NanoDrop One Spectrophotometer (Thermo Fisher), a Qubit RNA High Sensitivity (Invitrogen) and an Agilent 2100 Bioanalyzer system with an RNA nano chip. Library preparation and sequencing of small RNA samples were carried out by Vertis Biotechnologie (Freising). In brief, the small RNA samples were first treated with T4 polynucleotide kinase. Then oligonucleotide adapters were ligated to the 5′ and 3′ ends of the RNA samples. First-strand cDNA synthesis was done using M-MLV reverse transcriptase with the 3′ adapter as primer. The resulting cDNA was amplified with PCR using a high-fidelity DNA polymerase. The cDNA was purified using an Agencourt AMPure XP kit (Beckman Coulter Genomics) and was analysed by capillary electrophoresis. For Illumina NextSeq sequencing, the cDNAs were pooled in approximately equimolar amounts. The cDNA pool was purified using the Agencourt AMPure XP kit (Beckman Coulter Genomics) and was analysed by capillary electrophoresis. The primers used for PCR amplification were designed for TruSeq sequencing according to the instructions of Illumina. The NGS libraries (six samples) were single-read sequenced on an Illumina NextSeq 500 system using a read length of 75 bp at a depth of 10.2–11.5 million reads and were returned as sequences in FASTQ format.

### RNA-seq analysis

Generated reads in FASTQ format were initially processed by removing adaptors and low-quality reads using Trimmomatic^55^. The quality of the reads was assessed using FastQC v.0.11.9 (ref. ^56^) Processed reads were aligned to the *P.* *atrosepticum* (genome accession number BX950851.1) using Bowtie 2 (ref. ^57^) with local parameters and the alignment was converted to BAM format using SAMtools v.1.16.1 (ref. ^58^). The alignment was visualized and final images were generated using Geneious Prime 2022.1.1 (Dotmatics).

### RNA structure prediction

The RNA structures in Fig. 1a,b and Extended Data Figs. 1b, 2a,c,  3d, 4e,  9a and 10 were predicted using the RNAfold web server^59^ v.2.4.9 and visualized by RNA2Drawer^60^ v.6.3 and Adobe Illustrator v.27.

### 5′ RACE

To identify the 5′ end of the mRNA encoding RacrIF1 (experiment shown in Extended Data Figs. 1e and 3a) or RacrIC1 (experiment shown in Fig. 3e and Extended Data Fig. 9b,c), 5′ RACE was used to identify the 5′ end of the RNA transcript using the template-switching enzyme from NEB. In brief, RNA was extracted from overnight cultures in triplicate (for RacrIF1, PCF610 carrying an empty-vector control (pPF781) or the RacrIF1-expressing plasmid (pPF2845); for RacrIC1, POA1::IC carrying a plasmid expressing the Acr locus under wild-type promoter expression (pSC144)) using the Zymo-Seq RiboFree Total RNA Library Kit (Zymo Research). Afterwards, a template-switching reverse-transcription reaction was used to generate cDNAs with a universal sequence of choice (introduced by a template-switching oligonucleotide) attached to the 3′ end of the cDNA (the 5′ end of the transcript) (NEB). A sequence-specific reverse-transcription primer was placed so that it binds in the respective Racr or crRNA sequence. In the second step, the 5′ end of the transcript was identified by PCR amplification with primers that bind upstream from the Racr processing site and in the template-switching oligonucleotide, respectively. Oligonucleotides used are listed in Supplementary Table 3. PCR products were visualized on gels and cleaned up. For RacrIF1 under wild-type promoter expression, the size of the 5′ RACE product was visualized on a gel and analysed on a fragment analyser (experiment shown in Extended Data Fig. 1d), while the 5′ RACE product for RacrIC1 was visualized on a gel and sent for Sanger sequencing for confirmation (experiment shown in Extended Data Fig. 3b,c). 5′ RACE was also done to confirm the identity of the RNA species isolated by phenol/chloroform extraction and ethanol precipitation from the purified type I-F complex (Fig. 2c). PCR products were A-tailed with DreamTaq polymerase (Thermo Fisher) and dATP and cloned into pGEM-T Easy Vector (Promega). Plasmids were isolated from individual colonies and Sanger sequenced (Extended Data Fig. 3b,c).

### CRISPR-primed adaptation assay

The CRISPR adaptation assays shown in Fig. 2e and Extended Data Fig. 6b were performed as previously described^61^. A naive plasmid control (no matching protospacer, pPF953) and strong (AG PAM variant, pPF959) and medium (GT PAM variant, pPF967) priming-inducing plasmids were conjugated as described above (without Ara) into wild-type *P.* *atrosepticum* containing either a plasmid expressing RacrIF1 from the P_BAD_ promoter (pPF2846) or an empty-vector control (pPF781). The priming-inducing plasmids escaped targeting from the *P.* *atrosepticum* type I-F CRISPR–Cas system (Extended Data Fig. 6a). Strains with plasmids were grown in triplicate for 24 h in 5 ml LB supplemented with Cm and Tc. These ‘day 0’ cultures were then used to inoculate (1:500 dilution) 5 ml fresh LB supplemented with Cm, IPTG and Ara (without Tc selection), and incubated in the same conditions. This process was repeated for 5 days. Aliquots of culture from each day were mixed with 50% glycerol in a 1:1 ratio and frozen at −80 °C for future use. CRISPR array expansion (indicative of adaptation) was assessed by PCR using the cell glycerol stocks as a template. PCR products were loaded on a 2% agarose gel made up in 1× sodium borate buffer, run for 30 min at 180 V and stained with ethidium bromide.

### CRISPR-primed plasmid clearance assay

Plasmid clearance, visualized in Fig. 2f, was measured as previously described^35^. Cells from the CRISPR-primed adaptation assay (glycerol stocks) were diluted in 1 mL of PBS (1:1,000) and analysed using a BD LSRFortessa Cell Analyzer (BD Biosciences). A threshold was applied for FSC and SSC to detect bacterial cells. The mCherry was excited using a yellow–green laser (561 nm) and detected with a 610/20 nm bandpass filter; 20,000 events were recorded per sample using BD FACSDiva Software v.8 (BD Biosciences). Subsequent analysis was done using FlowJo Software v.10.8.1 (BD Biosciences). Cells were gated on SSC-A/SSC-H and SSC-A/FSC-A, then bifurcated (using BifurGate) into mCherry+ and mCherry− populations (Extended Data Fig. 6c). The ratio of mCherry− cells to total cells indicates the proportion of cells that cleared the plasmid.

### SRUFinder

We built a dedicated bioinformatic algorithm^40^ for finding SRU candidates in DNA sequences. The algorithm is available as a python package (https://pypi.org/project/srufinder) and a conda package (https://anaconda.org/russel88/srufinder) and is available at Zenodo. The algorithm is depicted as schematics in Extended Data Fig. 7. As queries, the algorithm uses a database of 17,823 non-redundant CRISPR repeat sequences with known associated subtypes (https://github.com/Russel88/SRUFinder/blob/master/data/repeats.fa). Repeats were obtained from the CCtyper^62^ web server (v. December 2020) and de-duplicated using cd-hit-env^63^ at 100% identity and coverage. First, open reading frames (ORFs) were predicted using prodigal^64^ in meta mode, and all ORFs with confidence ≥80% were masked from the input sequence. Next, repeat sequences were aligned with BLASTn^65^ against the masked input sequence with task = blastn − short and word size  =6. Matches with identity less than 90% were discarded. Furthermore, matches with coverage ≥90% were considered to be full matches, whereas matches with coverage between 50% and 90% were considered partial matches. If any alignments overlapped, only the match with the highest bit score was kept. Then all full matches within 100 bp were clustered into arrays, and these repeats were disregarded as potential SRUs. Furthermore, if a partial match was within 100 bp of a solitary full match, it was considered a mini-array if the identity between the two was ≥90% (biopython pairwise2.align.global, default match/mismatch penalties, −1 open/extend gap penalties, no end gap penalty)^66^. Then the remaining potential SRUs were aligned against the flanking 100 bp (biopython pairwise2.align.local, default match/mismatch penalties, −1 open/extend gap penalties). Because BLAST was observed to miss identifying repeats with several mismatches to the query, candidate SRUs showing partial matches (identity greater than 70%) to any of the two flanking regions (100 bp) were discarded to ensure that the SRUs were truly solitary. The remaining SRUs were then filtered by a bit score threshold of 41.1. This cut-off was set by running the algorithm on both intergenic (as described above) and intragenic (as above, but with ORF masking reversed) on the IMG/VR3 database^39^, and using recursive partitioning trees (rpart 4.1–15; ref. ^67^) to determine the best cut-off for distinguishing potential SRUs in intergenic regions (true candidates) from potential SRUs in intragenic regions (probably false-positive matches). We found that 84.0% of the matches with a bit score ≥41.1 were from intergenic regions, compared with 23.9% of matches with any bit score being from intergenic regions; 84.6% of matches with bit score of less than 41.1 were from intragenic regions.

### Bioinformatic search for SRUs in databases

Prophages were extracted using VIBRANT 1.0.1 (ref. ^68^) from the 104,858 high-quality genomes from GTDB (Version r95, 2020/10/06; ref. ^37^), which yielded 437,636 prophages from 69,688 of the genomes. SRUfinder^40^ was then run against these GTDB prophages, the PLSDB plasmid database (27,939 plasmid genomes^38^) and the IMG/VR3 database (2,332,702 virus genomes^39^). A virus dendrogram was created from the taxonomic information provided in the IMG/VR3 metadata and presented in Extended Data Fig. 8a. SRUFinder^40^ was also run against the PHASTER database (65,668 prophage and virus genomes^69^), but these SRUs were used only for finding candidates for experimental validation. The filtered output of SRUFinder^40^ can be accessed in Supplementary Data 1.

### GTDB prophage analysis

A phylogenetic tree of the GTDB-derived prophages containing SRUs was made by calling genes with prodigal^64^ and extracting 40 single-copy marker genes^70^ using fetchMGs 1.2 (https://github.com/motu-tool/fetchMGs). Each marker gene was then aligned separately with mafft 7.310 (ref. ^71^), the alignments were concatenated and a tree was inferred using FastTree 2.1.10 (ref. ^72^). Clades were collapsed with the collapse_tree_at_resolution function from R-package castor version 1.7.2 (ref. ^73^) at resolution 0.01 with rename_collapsed_nodes = TRUE. The tree was visualized with iTOL v.5 (ref. ^74^). Identification and subtyping of *cas* operons in the chromosomes was done with CCtyper 1.2.1 (ref. ^75^). To determine whether there was a non-random association between the subtype of the SRU and the subtype of any *cas* operon in the chromosome, we firstly restricted the analysis to SRUs where the host had any *cas* operon (that is, IMG/VR3 hits were excluded given the lack of known host associations). For this subset (170 SRUs of 188 total in GTDB prophages), 82.9% of the SRUs had a *cas* operon of matching subtype in the host. When the subtype of the SRU was permuted, there was a mean association of 32.3% with a standard deviation of 2.7% across 1,000 permutations. The data are illustrated in Extended Data Fig. 8b and the alignments of SRU sequences from strains with CRISPR–Cas, along with their corresponding consensus CRISPR repeats, are available in Supplementary Data 2.

### Association with *acr* genes

To establish whether *racr* candidates were co-located with *acr* genes, we used *acr* genes predicted by machine learning from ref. ^76^. Only predicted Acrs with a score greater than 0.5 were considered. Acr protein sequences were aligned against all virus and plasmid genomes containing SRUs with tblastn v.2.11.0+(ref. ^65^). Only matches with E-values ≤0.01 were kept. If matches were overlapping, only the match with the highest bit score was retained. To determine whether SRUs and *acr* genes are genetically co-located more often than random, the number of *acr* genes within 1 kb of an SRU was counted. This was then compared to the same statistic across 1,000 permutations in which the location of the SRU across the virus or plasmid genome was random. The data are depicted in Fig. 3c and a one-tailed *P* value was calculated as:

$$P=\frac{\left|acr\,{{\rm{within}}1{\rm{kb}}}_{{\rm{random}}} > acr\,{{\rm{within}}1{\rm{kb}}}_{{\rm{observed}}}\right|+1}{\mathrm{1,000}+1}$$

### Statistics and reproducibility

The specific test used for assessing statistical significance is indicated in the figure legends. The exact *P* values of the statistical analyses are stated in Supplementary Table 5. Protein purifications, RNA isolations and the phage infection assay on PAO1::I-C were independently repeated twice. Small RNA-seq, 5′ RACE, conjugation efficiency, primed adaptation and the phage infection assay with induction of Racr expression at different ALA concentrations were performed once with three independent biological replicates. All the other phage infection assays were independently repeated at least three times.

### Data visualization

Unless stated otherwise, data processing and visualization were done in Microsoft Excel v.16, Prism v.9.2.0 (GraphPad), SnapGene v.7.0.2 and Geneious Prime v.2022.1.1, and subsequently edited in Adobe Illustrator v.27. For gel source data, see Supplementary Fig. 1.

### Reporting summary

Further information on research design is available in the Nature Portfolio Reporting Summary linked to this article.

## Online content

Any methods, additional references, Nature Portfolio reporting summaries, source data, extended data, supplementary information, acknowledgements, peer review information; details of author contributions and competing interests; and statements of data and code availability are available at 10.1038/s41586-023-06612-5.

### Supplementary information

Supplementary Information The file contains Supplementary Fig. 1 and Supplementary Tables 1–5.

Reporting Summary

Supplementary Data 1 The file contains the filtered output of the SRUFinder in an editable format.

Supplementary Data 2 The file contains an alignment of SRU sequences found in strains with CRISPR–Cas and the according consensus CRISPR repeat in an editable format.

### Source data

Source Data Fig. 1

Source Data Fig. 2

Source Data Fig. 3

Source Data Extended Data Fig. 2

Source Data Extended Data Fig. 4

## Data Availability

Data that support the findings of this study are available within the paper and its Supplementary Information. Small RNA sequencing data is BioProject accession PRJNA893428 and BioSample accession SAMN31422748. We used the following datasets: PLSDB plasmid database (2020_11_19), IMG/VR3 database and PHASTER database. The database of 17,823 non-redundant CRISPR repeat sequences with known associated subtypes was made available here: https://github.com/Russel88/SRUFinder/blob/master/data/repeats.fa. Source data are provided with this paper.

## Code availability

The algorithm of SRUfinder is available as a Python package (https://pypi.org/project/srufinder) that is also a Conda package (https://anaconda.org/russel88/srufinder). The custom code was deposited in Zenedo^40^.
