# Supplementary material for: Bacteriophages suppress CRISPR–Cas immunity using RNA-based anti-CRISPRs
Source: Nature. 2023 Oct 18;623(7987):601–7. doi: 10.1038/s41586-023-06612-5 (PMC10651486; doi:10.1038/s41586-023-06612-5)
Supplement: Supplementary file 1 — The file contains Supplementary Fig. 1 and Supplementary Tables 1–5. [file 41586_2023_6612_MOESM1_ESM.pdf]

nature portfolio

https://doi.org/10.1038/s41586-023-06612-5

Supplementary information

Bacteriophages suppress CRISPR–Cas immunity using RNA-based anti-CRISPRs

In the format provided by the authors and unedited

Fig. 1f

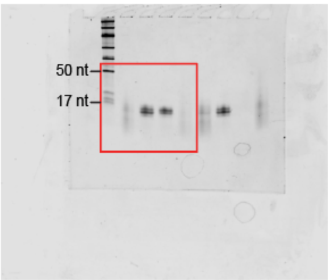

Fig. 2b

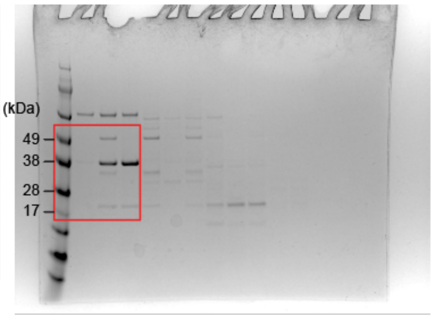

Fig. 2c

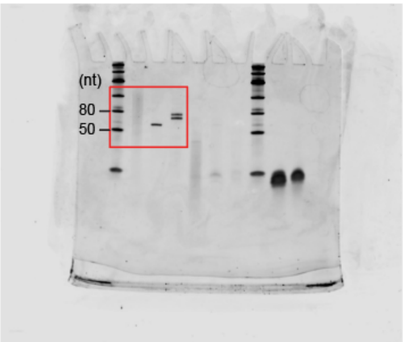

Fig. 2e and Extended Data Fig. 6b

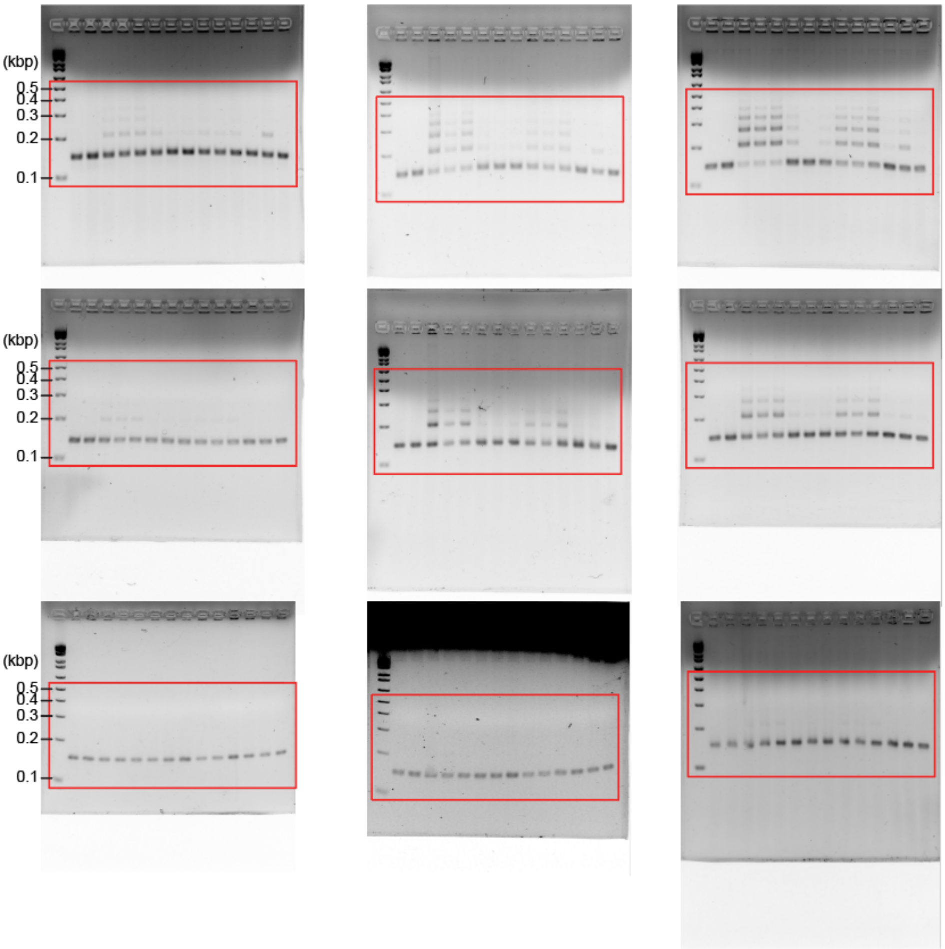

Fig. 3e and Extended Data Fig. 9b

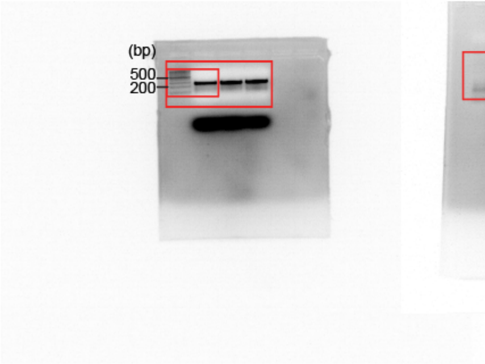

Extended Data Fig. 1e

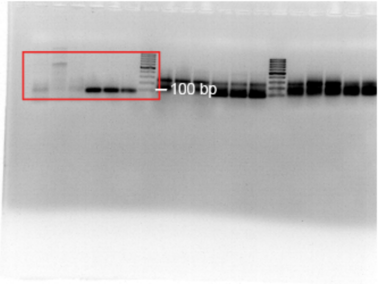

Extended Data Fig. 3a

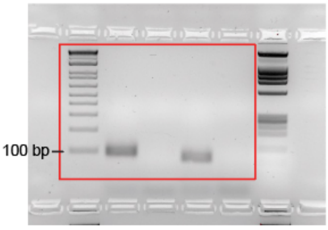

Supplementary Figure 1: Uncropped source gel images. Display of gel images. The red boxes indicate the cropping performed for display in the respective figure as indicated.

Supplementary Table 1: Information sheet of tested Racr candidates.

name	tested in strain	5'-3' sequence Racr candidate	5'-3' sequence model host consensus repeat	identity to model host repeat	host acc.	bacterial host	database	specification /alias
**Racr IF1**	Pba, PA14	GTTCACTGCCGGATAGGCAGCCAAGGAAA	GTTCACTGCCGTACAGGCAGCTTAGAAA (Pba); GTTCACTGCCGTATAGGCAGCTAAGAAA (PA14)	23/28 (Pba); 25/28 (PA14)	NC_018012.1	*Thiocystis violascens* DSM 198	PHASTER	Faure et al. (2019) [4] PPOA865
**Racr IF2**	Pba, PA14	CCTCACTGCCGTATAGGCAGCCCAGAAA	GTTCACTGCCGTACAGGCAGCTTAGAAA (Pba); GTTCACTGCCGTATAGGCAGCTAAGAAA (PA14)	22/28 (Pba); 23/28 (PA14)	CP011110.1	*Pseudomonas chlororaphis* strain PCL1606	PHASTER	SRUFinder finds part of it, lacking the first 2bp, found through manual curation
**Racr IF3**	Pba, PA14	GTTCACTGCCGCACAGGCAGCTTAAAAAT	GTTCACTGCCGTACAGGCAGCTTAGAAA (Pba); GTTCACTGCCGTATAGGCAGCTAAGAAA (PA14)	26/29 (Pba); 24/29 (PA14)	CP009866.1	*Pantoea sp.* PSNIH2	PHASTER	NZ_CP009866.1@1-4266503_2942379-2942407
**Racr IE1**	PA scm	GTGTTCCCCGCGTGTGCGGGGATGAACCG	GTGTTCCCCACGGGTGTGGGGATGAACC	25/29	CP011835.1	*Azotobacter chroococcum* strain B3	PHASTER	CP011835.1@1-4574769_601005-601033
**Racr IE2**	PA scm	GTGTTCCCCACGCACGTGGGGATGAACCG	GTGTTCCCCACGGGTGTGGGGATGAACC	25/29	MNPS01000007.1	*Saccharibacter sp.* M18	GTDB prophages	MNPS01000007.1_16441-16469
**Racr VA1**	PAO1::VA	GTCTAACGACCTTTTAAATTTCTACTGTTTGTAGAT	GTCTAACGACCTTTTAAATTTCTACTGTTTGTAGAT	36/36	CP011377.1	*Moraxella bovoculi* strain 23343	PHASTER	CP011377.1@256566-303249_6931-6966
**Racr VA2**	PAO1::VA	GTCTAACAACTTTTTAAATTTCTACTGTTTGTAGAT	GTCTAACGACCTTTTAAATTTCTACTGTTTGTAGAT	34/36	NZ_CP011376.1	*Moraxella bovoculi* strain 22581	PHASTER	NZ_CP011376.1@1313490-1335932_15419-15454
**Racr VA3**	PAO1::VA	GTCTAACGACTATTTAAATTTCTACTATTTGTAGAT	GTCTAACGACCTTTTAAATTTCTACTGTTTGTAGAT	33/36	NKHK01000012.1	*Moraxella sp.* VT-16-12	GTDB prophages	NKHK01000012.1_24241-24276
**Racr IC1**	PAO1::IC	GTCGCGCCCCGCGAGGGGGCGCGTGGATCGAAAC	GTCGCGCCCCGCACGGGCGCGTGGATTGAAAC	29/34	QRXC01000024.1	*Bifidobacterium pseudocatenulatum* strain AF18-2AC	GTDB prophages	QRXC01000024.1_2577-2610

Supplementary Table 2: Strains and phages used in this study.

name	genotype/phenotype	source	identifier
Pectobacterium atrosepticum			
SCRI1043	wild-type *Pba*	Bell et al. (2004) [80]	BX950851.1
PCF610	SCRI1043 with integrated T5/lac promoter for *cas* operon overexpression	Watson et al. (2019) [81]	
PCF188	SCRI1043 with 3x anti-ϕTE spacers (two in CRISPR1 and one in CRISPR2)	Pawluk, et al. (2016) [82]	
Escherichia coli			
DH5α	cloning strain. F- , φ80ΔdlacZM15, Δ(lacZYA–argF)U169, endA1, recA1, hsdR17 (rK- mK+), deoR, thi-1, supE44, λ-, gyrA96, relA1	Taylor et al. (1993) [83]	
ST18	auxotrophic donor for biparental conjugation. S17-1 λpir ΔhemA	Thoma et al. (2009) [84]<br>Jackson et al. (2020) [85]	
LOBSTR	protein expression strain. Str., B, F–, ompT, gal, dcm, lon, hsdSB(rB– mB–), λ(DE3, [lacI, lacUV5-T7p07, ind1, sam7, nin5]), [malB+]K-12(λS), arnA, slyD	Kerafast	
GeneHogs(R)		Thermofisher, Catalog no. C8080-10	
Pseudomonas aeruginosa			
PA14	WT	Lee et al. (2006) [86]	NC_008463.1
PA14	UCBPP-PA14 ΔCRISPR1ΔCRISPR2 (SMC5454)	Cady et al. (2012) [87]	
SMC4386	WT	Cady et al. (2011) [88]	LOQZ00000000
SMC4386	ΔCas3	Cady et al. (2012) [87]	
PAO1	WT	Windsor et al. (2009) [89]	NC_002516.2
PAO1::IC	I-C CRISPR-Cas (LL77)	Marino et al. (2018) [90]	
PAO1::VA	tn7::mbCpf1, ctx2::crRNA23	Marino et al. (2018) [90]	
PAO1::VA - crRNA	tn7::mbCpf1, ctx2:: no crRNA	Marino et al. (2018) [90]	
**Bacteriophages**			
Pectobacterium phage ϕTE		Blower et al. (2012) [91]	
Pseudomonas phage DMS3m		Cady et al. (2012) [87]	
Pseudomonas phage JBD30		Bondy-Denomy et al. (2013) [3]	

Supplementary Table 3: Oligonucleotides used in this study.

name	sequence (5′-3′)	description
**cloning**		
PF5520	ATA**GGTCTC**ATGGAGAAACAGTAGAGAGTTG	R primer to introduce a BsaI restriction site in pPF781 downstream of P_{BAD}
PF5564	ATA**GGTCTC**GAATTCGAGCTCGGTACC	F primer to introduce a BsaI restriction site in pPF781 upstream of the terminator
PF5565	ATA**GGTCTC**CTCCATTGGATTTGAACGGTTCACTGC	F primer for cloning RacrIF3 into pPF781, generating pPF2802 (BsaI)
PF5566	ATA**GGTCTC**GAATTCTTCGATTTCTTCTTTATTCAGCAGGATG	R primer for cloning RacrIF3 into pPF781, generating pPF2802 (BsaI)
PF5567	ATA**GGTCTC**CTCCATTGGTGTTGCTGGACTACCTGTCC	F primer for cloning RacrIF2 into pPF781, generating pPF2803 (BsaI)
PF5568	ATA**GGTCTC**GAATTCTGATACGATTAGGACAATGGTCACCGA	R primer for cloning RacrIF1 or RacrIF2 into pPF781, generating pPF2845 (RacrIF1-pWT), pPF2846 (RacrIF1-P_{BAD}) or pPF2803 (RacrIF3-P_{BAD}) (BsaI)
PF5658	ATA**GGTCTC**GCTTTGTTACAGAATGCTTTTAATAAGC	R primer to introduce BsaI restriction sites in pPF781 upstream of P_{BAD}
PF5661	ATA**GGTCTC**CAAAGCGAGACAAGGTCGCCTTGTCTCG	F primer for cloning RacrIF1-pWT into pPF781, generating pPF2845 (BsaI)
PF5662	ATA**GGTCTC**CTCCATGTCGCCCGATTGCGCGA	F primer for cloning RacrIF1 into pPF781, generating pPF2846 (BsaI)
PF5663	GCCGGATAGGCACCCAAGG	F primer for site-directed mutagenesis of type I-F SRU C6G/G20C in pPF2845 or pPF2846, generating pPF2895 or pPF2847, respectively
PF5664	TGCCTATCCGGCACTGAACG	R primer for site-directed mutagenesis of type I-F SRU C6G/G20C in pPF2845 or pPF2846, generating pPF2895 or pPF2847, respectively
PF5314	CATCACCATCACCATCACGGATCCATGGATCACTACATTGATAT	F primer to introduce a 6xHis-tag in the N-term of Cas6f
PF5315	GTGATGGTGATGCGATCCTCTCATATGGTATATCTCCTTATTAAAG	R primer to introduce a 6xHis-tag in the N-term of Cas6f
PF5703	ATA**GGTCTC**TAGGCTGCTGCCACC	F primer to introduce a BsaI restriction site in pPF2640 upstream of the terminator
PF5704	ATA**GGTCTC**CCTATAGTGAGTCGTATTAATTTCGATTATG	R primer to introduce a BsaI restriction site in pPF2640 downstream of T7 promoter
PF5705	ATA**GGTCTC**TATAGGGTTCACTGCCGTACAGGCAGCTTAGAAAGCAGAGACTATCGATACGGTCTGGAC	F primer for cloning the Pba type I-F crRNA (repeat-spacer-repeat) into pPF2640, generating pPF2644 (BsaI)
PF5706	ATA**GGTCTC**AGCCTATTTCTAAGCTGCCTGTACGGCAGTGAACGCATCCGTCCAGACCGTATCGATAGT	R primer for cloning the Pba type I-F crRNA (repeat-spacer-repeat) into pPF2640, generating pPF2644 (BsaI)
PF5707	ATA**GGTCTC**TATAGGGTCGCCCGATTGCGCGA	F primer for cloning RacrIF1 into pPF2640, generating pPF2868 (BsaI)
PF5708	ATA**GGTCTC**AGCCTATGATACGATTAGGACAATGGTCACCGA	R primer for cloning RacrIF1 into pPF2640, generating pPF2868 (BsaI)
PF7342	TCGTCTTCACCTCGAGAAATCGGTCTCCTCCATCCTTGGCTGATGAGTCCGTGAGGACGAAACGAGTAAGCTCGTCCCAAGGAAATCATGCTCGGCCACGGACGGTCTTCCCCCTGCGCCCCCGCAACCTATTGTGCACATCGCCCGCCGTCGATCATGGCGGCCGTCGGTGACCATTGTCCTAATCGTATCAGAATTCGAGACCCCTGTTGATAGATCCAGTAATGAC	RacrIF1 no stem-loop and 5' hammerhead ribozyme with overhangs containing 6 bp matching downstream P_{BAD} promoter in pPF781 and BsaI restriction sites. BsaI digest and ligate into BsaI digested pPF781, generating pPF3600
PF7348	AGTTCCTTGGATCATGCTCGGCCAC	F primer to substitute RacrIF1 5' handle for the reverse complement. 5' overlaps 12 bp with 5' PF7347. Combine in a PCR of pPF2846, generating pPF3604
PF7349	ATCCAAGGAACTGCCTATCCGGCAG	R primer to substitute RacrIF1 5' handle for the reverse complement. 5' overlaps 12 bp with 5' PF7346. Combine in a PCR of pPF2846, generating pPF3604
PF3250	TTTTCAATTGAGGAGGAATTAACATGAGAAATGGACTACCCGAATTC	F primer for cloning the *Pba csy1-3* operon into pQE-80L-oriT, generating pPF1635 (MfeI + RBS). Binds to *csy1* start codon
PF3251	TTTTCTGCAGTTATTCGCCTTTTTCACCAAACAC	R primer for cloning the *Pba csy1-3* operon into pQE-80L-oriT, generating pPF1635 (PstI). Binds to *csy3* stop codon
Prs216	AGGCAGCUTAGAAATGAGACCTG	F primer to clone pecto type I-F repeat-BsaI-repeat from pPF975 on pSC386
Prs217	ACCGAGCUCGCATGCTTTC	R primer to clone pecto type I-F repeat-BsaI-repeat from pPF975 on pSC386
Prs215	AGCTCGGUGAATTCGAGC	F primer to amplify backbone of pF178 to clone pecto type I-F repeat-BsaI-repeat into it, resulting in pSC386

Prs214	AGCTGCCUGTACGGCAGTGAACTGG AGAAACAGTAGAGAGTTGC	R primer to amplify backbone of pF178 to clone pecto type I-F repeat-BsaI-repeat into it, resulting in pSC386
Prs220	ACTGCCGGAUAGGCAGCCAAGGAAT GAGACCTGCTG	F Primer replacing first repeat in pSC386 with SRU865, resulting in pSC387
Prs221	ATCCGGCAGUGAACTGGAGAAAC	R Primer replacing first repeat in pSC386 with SRU865, resulting in pSC387
Ors132	GAAATGACACAGCCAACGCCCTGAA AATCGGCACAGG	Oligo annealed with Ors134 for spacer ØTE insertion into pSC386, resulting in pSC391
Ors133	GGAATGACACAGCCAACGCCCTGAA AATCGGCACAGG	Oligo annealed with Ors134 for spacer ØTE insertion into pSC387, resulting in pSC392
Ors134	TGAACCTGTGCCGATTTTCAGGGCG TTGGCTGTGTCA	Oligo annealed with Ors132 or Ors133 for spacer ØTE insertion into pSC386 or pSC387 respectively, resulting in pSC391 and pSC392 respectively
Prs31a	ACTGTTTCUCCATCCGGGGCCTGCT CTC	F primer for mamplification of RacrIF1 to clone behind P_{BAD} +1
Prs78	ACTGTTTCUCCATTGGTGTTGCTGG ACTACCTG	F primer for amplification of RacrIF1 to clone behind P_{BAD} +1 amplification of RacrIF2 to clone behind P_{BAD} +1
Prs80	ACTGTTTCUCCATTGGATTTGAACGG TTCACTGCCG	F primer for amplification of RacrIF3 to clone behind P_{BAD} +1
Prs65	ACTGTTTCUCCATCCGTGTTCCCCG CGTGTGC	F primer for amplification of RacrIE1 to clone behind P_{BAD} +1
Prs112	ACTGTTTCUCCATCCGCCCTCTCTGT CGTGGAAG	F primer for amplification of RacrIE2 to clone behind P_{BAD} +1
Prs55	ACTGTTTCUCCATTGGTCGCATCACA GCAAATAG	F primer for amplification of RacrVA1 to clone behind P_{BAD} +1
Prs54	ACTGTTTCUCCATTGTTTTTAAACCA TGTCAATTG	F primer for amplification of RacrVA2 to clone behind P_{BAD} +1
Prs107	ACTGTTTCUCCATTTTTTTAAAATGG TGAAAGTCTAAC	F primer for amplification of RacrVA3 to clone behind P_{BAD} +1
Scp14	AGTCCGAUCCCAACTATTTTGTCCG CCCAC	F amplification of Racr candidates from twist fragments
Prs108	ACCCAUGAGCACCATCATCGACCAG GAC	F primer for amplification of AcrIC5 homologue and RacrIC1 behind P_{BAD} and RBS
Prs9	ACGGCCAGUTGATACGATTAGGACA ATGGTCACCGACG	R amplification of Racr candidates from twist fragments
Prs11	ACGGCCAGUTAGCCTGAGGGACTAA GGGAAACAGTTGTC	R amplification of Racr candidates from twist fragments
Prs83	ACGGCCAGUATCTACAAACAGTAGA AATTTAAAAAG	R primer for amplification of RacrVA2
Prs111	ACGGCCAGUCGCCGTGCTTGTCAGA TGGTG	R primer amplification of acr locus without RacrIC1 behind P_{BAD}
Prs120	ACCGCCGUGGCGTTAGTCGATTT	Introduction of frameshift into acrIC5
Prs121	ACGGCGGUCAACCTCATGGACG	Introduction of frameshift into acrIC5
Prs1a	AGAAACAGUAGAGAGTTGCGATAAA AAGCGTCAG	pHerd-30T backbone amplification
Prs2	ACTGGCCGUCGTTTTACAACGTCG	pHerd-30T backbone amplification
Prs8	ATCGGACUGCTTTGTTACAGAATGC TTTTA	pHerd-30T backbone amplification
Prs109	ATGGGUATGTATATCTCCTTCTTAAA GTTAAAC	pHerd-30T backbone amplification
Prs4	GCTGCAAGGCGATTAAGTTGG	Sanger sequencing cloning site pHerd-30T
Prs212	AACGAAUCAGACAATTGACGG	R primer to remove P_{BAD} and AraC and clone RacrIF1 behind different promoters used on pPF2846
Prs213	AGCGUCGCCCGATTGCGC	F primer to remove P_{BAD} and AraC and clone RacrIF1 behind different promoters used on pPF2846
Ors124	AGCATAATCCCTAGGACTGAGCTAG CTATCAGAACGAAT	Oligo annealed with Ors125 for insertion of promoter BBa_J23112 in front of RacrIF1
Ors125	CTGATAGCTAGCTCAGTCCTAGGGA TTATGCTAGCGT	Oligo annealed with Ors124 for insertion of promoter BBa_J23112 in front of RacrIF1
Ors128	AGCATTGTACCTAGGACTGAGCTAG CCGTAAAAACGAAT	Oligo annealed with Ors129 for insertion of promoter BBa_J23110 in front of RacrIF1
Ors129	TTTACGGCTAGCTCAGTCCTAGGTA CAATGCTAGCGT	Oligo annealed with Ors128 for insertion of promoter BBa_J23110 in front of RacrIF1

Ors130	AGCACTGTACCTAGGACTGAGCTAG CCGTCAAAACGAAT	Oligo annealed with Ors131 for insertion of promoter BBa_J23100 in front of RacrIF1
Ors131	TTGACGGCTAGCTCAGTCCTAGGTA CAGTGCTAGCGT	Oligo annealed with Ors130 for insertion of promoter BBa_J23100 in front of RacrIF1
**CRISPR array expansion**		
PF174	CGTTAGAGTGATCGGGCTAC	F primer for Pba CRISPR1 (binds in leader)
PF175	CAATGGCTCAGGGGATTC	R primer for Pba CRISPR1 (binds in spacer 2)
PF176	GGTAACTACCGTAAAATAGGAACG	F primer for Pba CRISPR2 (binds in leader)
PF177	GCCTTTAAGCGCATGTCG	R primer for Pba CRISPR2 (binds in spacer 2)
PF178	CTTTAATAATCTGGTTGTTAGTGTG	F primer for Pba CRISPR3 (binds in leader)
PF179	CCTCAGAAAGCCGACTTC	R primer for Pba CRISPR3 (binds in spacer 2)
**screening**		
PF138	CACACTTTGCTATGCCATAG	pPF781-derived plasmids, F primer cloning site
PF1702	CGAAGACGAAAGGGCCTCGTGATAC GCAAGCTTTATGGCTTGTAAACCGTT TTGTG	pPF781-derived plasmids, R primer cloning site
PF2026	GGATCTCGACGCTCTCCCTT	6xHis-tag insertion in pPF2640, F primer
PF757	GCTCTAGAGGGCCATGTTCCACAAA CAC	6xHis-tag insertion in pPF2640, R primer
PF2084	TTGTACACGGCCGCATAATC	pPF2640-derived plasmids, F primer cloning site
PF1641	GCTAGTTATTGCTCAGCGG	pPF2640-derived plasmids, R primer cloning site
PF1218	GATGTCAAAACCGTCAAAGAG	*Pba* gDNA contamination check, F primer
PF1219	TTCTTGTACTGGTCGCGTTC	*Pba* gDNA contamination check, R primer
**5' RACE**		
TSO	GCTAATCATTGCAAGCAGTGGTATC AACGCAGAGTACATrGrGrG	Template switching oligo used for 5' RACE (NEB)
TSO-PCR	CATTGCAAGCAGTGGTATCAAC	F primer for amplification of 5' RACE product
RT-RacrIF1	ATGCAGCTACGACCTATCCGGCAGT GAACGAGAGC	reverse transcription primer binding in the RacrIF1 candidate sequence
RT-PCR-RacrIF1	ACGAGAGCATCAGACAAGGCGGC	R primer for amplification of 5' RACE product of RacrIF1
RT-crRNA	ATGCAGCTACGACCTGTACGGCAGT GAACGCATCC	reverse transcription primer binding in the crRNA
RT-PCR-crRNA	ACGCATCCGTCCAGACCGTATCG	R primer for amplification of 5' RACE product of the crRNA
RT-RacrIC1	ATGCAGCTACGATCGCGGGGCGCG ACCGCCG	reverse transcription primer binding in the RacrIC1 candidate sequence
RT-PCR-RacrIC1	GATCGCGGGGCGCGACCG	R primer for amplification of 5' RACE product of RacrIC1 and for sanger sequencing of the PCR product
PF861	TAATACGACTCACTATAGGG	primer for sequencing of 5' RACE products cloned into pGEM-T

Supplementary Table 4: Plasmids used in this study.

name	description	features	construction	reference
**phage targeting (Fig. 1)**				
pPF975	pecto type I-F repeat-BsaI-repeat construct for IPTG-inducible crRNA overexpression	pBR322/ori, RP4/oriT, KmR, lacI/T5	pMAT16 derivative	Jackson et al. 2019 [35]
pPF1423	anti-ϕTE spacer overexpression (type I-F)	pBR322/ori, RP4/oriT, KmR, lacI/T5	pPF975 derivative	Watson et al. 2019 [81]
**Racr candidates expression (Figs. 1 and 3)**				
pPF781	empty vector control for expression	p15A/ori, RP4/oriT, CmR, P_{BAD}/araC	P_{BAD}30 derivative	Patterson et al. 2016 [61]
pRS-I-F-1	template for RacrIF1 cloning into expression vector	ori1600 pBR322 GmR OriT araC	Prs8 and Prs2 paired in a PCR to amplify the backbone and to introduce a Uracil for USER cloning downstream of P_{BAD}. Scp14 and Prs9 paired in a PCR to amplify the I-F SRU with wildtype promoter from twist fragment and to introduce a Uracil for USER cloning.	This study
pPF2845	RacrIF1-pWT expression	p15A/ori, RP4/oriT, CmR	PF5564 + PF5658 paired in a PCR to introduce BsaI restriction sites in pPF781 upstream of P_{BAD} and upstream of the terminator. PF5661 + PF5568 paired in a PCR to amplify RacrIF1 from pRS-I-F-1. Both PCR products were digested with BsaI and DpnI, and ligated.	This study
pPF2895	RacrIF1-pWT expression: stem-loop C6G/G20C mutant	p15A/ori, RP4/oriT, CmR	PF5663 + PF5664 paired in a PCR to introduce *RacrIF1* C6G/G20C mutation in pPF2845	This study
pPF2846	RacrIF1-P_{BAD} expression	p15A/ori, RP4/oriT, CmR, P_{BAD}/araC	PF5564 + PF5520 paired in a PCR to introduce BsaI restriction sites in pPF781 downstream of P_{BAD} and upstream of the terminator. PF5662 + PF5568 paired in a PCR to amplify RacrIF1 from pRS-I-F-1. Both PCR products were digested with BsaI and DpnI, and ligated.	This study
pPF2847	RacrIF1-P_{BAD} expression: stem-loop C6G/G20C mutant (Variant 1)	p15A/ori, RP4/oriT, CmR, P_{BAD}/araC	PF5663 + PF5664 paired in a PCR to introduce *RacrIF1* C6G/G20C mutation in pPF2846	This study
pPF2802	RacrIF3-P_{BAD} expression	p15A/ori, RP4/oriT, CmR, P_{BAD}/araC	PF5564 + PF5520 paired in a PCR to introduce BsaI restriction sites in pPF781 downstream of P_{BAD} and upstream of the terminator. PF5565 + PF5566 paired in a PCR to amplify RacrIF3 from pSC081. Both PCR products were digested with BsaI and DpnI, and ligated.	This study
pPF2803	RacrIF2-P_{BAD} expression	p15A/ori, RP4/oriT, CmR, P_{BAD}/araC	PF5564 + PF5520 paired in a PCR to introduce BsaI restriction sites in pPF781 downstream of P_{BAD} and upstream of the terminator. PF5567 + PF5568 paired in a PCR to amplify RacrIF2 from pSC013. Both PCR products were digested with BsaI and DpnI, and ligated.	This study
pPF3600	RacrIF1-P_{BAD} expression: stem-loop knockout, hammerhead ribozyme 5' handle (Variant 3)	p15A/ori, RP4/oriT, CmR, P_{BAD}/araC	PF5564 + PF5520 paired in a PCR to introduce BsaI restriction sites in pPF781. Digest product with BsaI (and DpnI to cleave template) and ligate into BsaI digested PF7342 gBlock	This study
pPF3604	RacrIF1-P_{BAD} expression: 5' handle reverse complement mutation (Variant 2)	p15A/ori, RP4/oriT, CmR, P_{BAD}/araC	PF7348 + PF7349 paired in a PCR to substitute *RacrIF1* 5' handle for the reverse complement in pPF2846	This study
pSC373	RacrIF1 expression under biobrick promoter BBa_J23100	p15A/ori, RP4/oriT, CmR	Prs212 and Prs213 paired in a PCR to amplify the backbone excluding the P_{BAD} promoter and the AraC and to introduce a Uracil for USER cloning. Afterwords annealed oligos Ors130/131 with according overhangs containing the promoter element were inserted via USER cloning	This study

pSC374	RacrIF1 expression under biobrick promoter BBa_J23110	p15A/ori, RP4/oriT, CmR	Prs212 and Prs213 paired in a PCR to amplify the backbone excluding the P_{BAD} promoter and the AraC and to introduce a Uracil for USER cloning. Afterwords annealed oligos Ors128/129 with according overhangs containing the promoter element were inserted via USER cloning	This study
pSC375	RacrIF1 expression under biobrick promoter BBa_J23112	p15A/ori, RP4/oriT, CmR	Prs212 and Prs213 paired in a PCR to amplify the backbone excluding the P_{BAD} promoter and the AraC and to introduce a Uracil for USER cloning. Afterwords annealed oligos Ors124/125 with according overhangs containing the promoter element were inserted via USER cloning	This study
pSC386	expression of canonical crRNA non-targeting	p15A/ori, RP4/oriT, CmR, P_{BAD}/araC	Prs216 + Prs217 paired in a PCR to amplify the backbone of pPF781 and to introduce a Uracil for USER cloning. Prs216 + Prs217 paired in PCR to amplify pecto type I-F repeat-BsaI-repeat from pPF975	This study
pSC387	hybrid crRNA non-targeting cloning intermediate	p15A/ori, RP4/oriT, CmR, P_{BAD}/araC	Prs220 + Prs221 paired in PCR on pSC386 to replace the first pecto repeat with SRU865	This study
pSC391	expression of canonical crRNA targeting ØTE	p15A/ori, RP4/oriT, CmR, P_{BAD}/araC	pSC386 was BsaI restricted, Ors132 + Ors134 were annealed and inserted	This study
pSC392	expression of hybrid crRNA targeting ØTE	p15A/ori, RP4/oriT, CmR, P_{BAD}/araC	pSC387 was BsaI restricted, Ors132 + Ors133 were annealed and inserted	This study
pHerd30t	empty vector control for expression	ColE/RepB oriR; RP4 oriT, GentR, P_{BAD}/araC	N/A	Qiu et al. 2008 [92]
pSC015	RacrIF1-P_{BAD} expression in PA14	ColE/RepB oriR; RP4 oriT, GentR, P_{BAD}/araC	Prs1a and Prs2 paired in a PCR to amplify the backbone and to introduce a Uracil for USER cloning downstream of P_{BAD}. Prs31a and Prs9 paired in a PCR to amplify the Racr candidate from twist fragment and to introduce a Uracil for USER cloning.	This study
pSC013	RacrIF2-P_{BAD} expression in PA14	ColE/RepB oriR; RP4 oriT, GentR, P_{BAD}/araC	Prs1a and Prs2 paired in a PCR to amplify the backbone and to introduce a Uracil for USER cloning downstream of P_{BAD}. Prs78 and Prs9 paired in a PCR to amplify the Racr candidate from twist fragment and to introduce a Uracil for USER cloning.	This study
pSC081	RacrIF3-P_{BAD} expression in PA14	ColE/RepB oriR; RP4 oriT, GentR, P_{BAD}/araC	Prs1a and Prs2 paired in a PCR to amplify the backbone and to introduce a Uracil for USER cloning downstream of P_{BAD}. Prs80 and Prs11paired in a PCR to amplify the Racr candidate from twist fragment and to introduce a Uracil for USER cloning.	This study
pSC011	RacrIE1-P_{BAD} expression in PAscm	ColE/RepB oriR; RP4 oriT, GentR, P_{BAD}/araC	Prs1a and Prs2 paired in a PCR to amplify the backbone and to introduce a Uracil for USER cloning downstream of P_{BAD}. Prs65 and Prs9 paired in a PCR to amplify the Racr candidate from twist fragment and to introduce a Uracil for USER cloning.	This study
pSC143	RacrIE2-P_{BAD} expression in PAscm	ColE/RepB oriR; RP4 oriT, GentR, P_{BAD}/araC	Prs1a and Prs2 paired in a PCR to amplify the backbone and to introduce a Uracil for USER cloning downstream of P_{BAD}. Prs112 and Prs9 paired in a PCR to amplify the Racr candidate from twist fragment and to introduce a Uracil for USER cloning.	This study
pSC144	WT promoter expression AcrIC5 + RacrIC1 in PAO1::IC	ColE/RepB oriR; RP4 oriT, GentR, araC	Prs8 and Prs2 paired in a PCR to amplify the backbone and to introduce a Uracil for USER cloning downstream of P_{BAD}. Scp14 and Prs9paired in a PCR to amplify the acr locus with wildtype promoter from twist fragment and to introduce a Uracil for USER cloning.	This study
pSC145	P_{BAD} expression AcrIC5 + RacrIC1 in PAO1::IC	ColE/RepB oriR; RP4 oriT, GentR, P_{BAD}/araC	Prs109 and Prs2 paired in a PCR to amplify the backbone and to introduce a Uracil for USER cloning downstream of P_{BAD}. Prs108 and Prs9 paired in a PCR to amplify the acr locus from twist fragment and to introduce a Uracil for USER cloning.	This study
pSC146	P_{BAD} expression AcrIC5 - RacrIC1 in PAO1::IC	ColE/RepB oriR; RP4 oriT, GentR, P_{BAD}/araC	Prs109 and Prs2 paired in a PCR to amplify the backbone and to introduce a Uracil for USER cloning downstream of P_{BAD}. Prs108 and Prs111paired in a PCR to amplify the acr	This study

			locus without the Racr candidate from twist fragment and to introduce a Uracil for USER cloning.	
pSC202	P_{BAD} expression AcrIC5(frameshift) + RacrIC1 in PAO1::IC	ColE/RepB oriR; RP4 oriT, GentR, P_{BAD}/araC	Prs120 and Prs121paired in a PCR on pSC145 to introduce frameshift in acrIC5	This study
pSC201	P_{BAD} expression AcrIC5(frameshift) - RacrIC1 in PAO1::IC	ColE/RepB oriR; RP4 oriT, GentR, P_{BAD}/araC	Prs120 and Prs121 paired in a PCR on pSC146 to introduce frameshift in acrIC5	This study
pSC149	RacVA3-P_{BAD} expression in PAO1::VA	ColE/RepB oriR; RP4 oriT, GentR, P_{BAD}/araC	Prs1a and Prs2 paired in a PCR to amplify the backbone and to introduce a Uracil for USER cloning downstream of P_{BAD}. Prs107 and Prs9 paired in a PCR to amplify the Racr candidate from twist fragment and to introduce a Uracil for USER cloning.	This study
pSC026	RacVA1-P_{BAD} expression in PAO1::VA	ColE/RepB oriR; RP4 oriT, GentR, P_{BAD}/araC	Prs1a and Prs2 paired in a PCR to amplify the backbone and to introduce a Uracil for USER cloning downstream of P_{BAD}. Prs55 and Prs9paired in a PCR to amplify the Racr candidate from twist fragment and to introduce a Uracil for USER cloning.	This study
pSC088	RacVA2-P_{BAD} expression in PAO1::VA	ColE/RepB oriR; RP4 oriT, GentR, P_{BAD}/araC	Prs1a and Prs2 paired in a PCR to amplify the backbone and to introduce a Uracil for USER cloning downstream of P_{BAD}. Prs54 and Prs83 paired in a PCR to amplify the Racr candidate from twist fragment and to introduce a Uracil for USER cloning.	This study
**conjugation efficiency, primed adaptation and plasmid clearance assays (Figs. 1 and 2)**				
pPF953	Untargeted control - lacks any protospacer	pBR322/ori, RP4/oriT, TcR, lacI/T5, mCherry	pQE-80L-oriT derivative	Jackson et al. 2019 [35]
pPF954	GGA, *Pba* type I-F canonical target	pBR322/ori, RP4/oriT, TcR, lacI/T5, mCherry	pPF953 derivative	Jackson et al. 2019 [35]
pPF959	AGA PAM variant	pBR322/ori, RP4/oriT, TcR, lacI/T5, mCherry	pPF953 derivative	Jackson et al. 2019 [35]
pPF967	GTA PAM variant	pBR322/ori, RP4/oriT, TcR, lacI/T5, mCherry	pPF953 derivative	Jackson et al. 2019 [35]
**protein and RNA expression (Figs. 1 and 2)**				
pPF2640	*Pba* His6-Cas6f expression	RSF1030/ori, KmR, lacI/T7	pRSF-1b derivative. PF5314 + PF5315 paired in a site-directed mutagenesis PCR of pPF2465	This study
pPF2644	*Pba* His6-Cas6f and type I-F crRNA repeat-spacer-repeat expression	RSF1030/ori, KmR, lacI/T7	PF5703 + PF5704 paired in a PCR to introduce BsaI restriction sites in pPF2640 downstream of T7 promoter and upstream of the terminator. PF5705 + PF5706 paired in a PCR to amplify the Pba type I-F crRNA (repeat-spacer-repeat). Both PCR products were digested with BsaI and DpnI, and ligated.	This study
pPF2868	*Pba* His6-Cas6f and RacrIF1 expression	RSF1030/ori, KmR, lacI/T7	PF5703 + PF5704 paired in a PCR to introduce BsaI restriction sites in pPF2640 downstream of T7 promoter and upstream of the terminator. PF5707 + PF5708 paired in a PCR to amplify RacrIF1 from pPF2846. Both PCR products were digested with BsaI and DpnI, and ligated.	This study
pPF2869	*Pba* His6-Cas6f and RacrIF1-M1 expression	RSF1030/ori, KmR, lacI/T7	PF5663 + PF5664 paired in a PCR to introduce *RacrIF1* C6G/G20C mutation in pPF2868	This study
pPF1635	*Pba* Cas8f-Cas5f-Cas7f untagged expression	pBR322/ori, RP4/oriT, ApR, lacI/T5	PF3250 + PF3251 paired in a PCR to amplify *csy1-3* operon from *Pba*. The PCR product and vector pQE-80L-oriT were digested with MfeI and PstI, and ligated	This study
**5' RACE (Extended Data Fig. 3)**				

pGEM®-T Easy Vector	vector for cloning the 5' RACE products originating from the RNA species purified from the type I-F protein complex	f1/ori, ApR, *lacZ*		Promega

Supplementary Table 5: Overview of p-values retrieved from statistical analyses.

figure	panel	sample	p-value
1	d	+RacrIF1	0.0165
1	d	+RacrIF1-GCmut	0.8585
1	e	+RacrIF1	<0.0001
2	f, –RacrIF1	strong priming, AG PAM variant	<0.0001
2	f, –RacrIF1	medium priming, GT PAM variant	0.0001
2	f, +RacrIF1	strong priming, AG PAM variant	0.3257
2	f, +RacrIF1	medium priming, GT PAM variant	>0.9999
3	c	GTDB*	0.018
3	c	IMG/VR*	<0.001
3	c	PLSDB*	0.53
3	f	acrIC5 wild-type, -RacrIC1	<0.0001
3	f	acrIC5 wild-type, +RacrIC1	<0.0001
3	f	acrIC5 truncated, -RacrIC1	0.3384
3	f	acrIC5 truncated, +RacrIC1	<0.0001
3	g	Pba, RacrIF2	0.0813
3	g	Pba, RacrIF3	0.0004
3	g	PA14, RacrIF1	<0.0001
3	g	PA14, RacrIF2	0.0008
3	g	PA14, RacrIF3	<0.0001
3	g	PAsmc, RacrIE1	<0.0001
3	g	PAsmc, RacrIE2	<0.0001
3	g	PAO1::V-A, RacrVA1	0.0006
3	g	PAO1::V-A, RacrVA2	0.0002
3	g	PAO1::V-A, RacrVA3	0.0335

* note that the p-value is based on 1000 random samplings

References

80. Bell, K. S. *et al.* Genome sequence of the enterobacterial phytopathogen Erwinia carotovora subsp. atroseptica and characterization of virulence factors. *Proc. Natl. Acad. Sci. U. S. A.* **101**, 11105–11110 (2004).

81. Watson, B. N. J. *et al.* Type I-F CRISPR-Cas resistance against virulent phages results in abortive infection and provides population-level immunity. *Nat. Commun.* **10**, 5526 (2019).

82. Pawluk, A. *et al.* Inactivation of CRISPR-Cas systems by anti-CRISPR proteins in diverse bacterial species. *Nat Microbiol* **1**, 16085 (2016).

83. Taylor, R. G., Walker, D. C. & McInnes, R. R. E. coli host strains significantly affect the quality of small scale plasmid DNA preparations used for sequencing. *Nucleic Acids Res.* **21**, 1677–1678 (1993).

84. Thoma, S. & Schobert, M. An improved Escherichia coli donor strain for diparental mating. *FEMS Microbiol. Lett.* **294**, 127–132 (2009).

85. Jackson, S. A., Fellows, B. J. & Fineran, P. C. Complete Genome Sequences of the Escherichia coli Donor Strains ST18 and MFD. *Microbiol Resour Announc* **9**, (2020).

86. Lee, D. G. *et al.* Genomic analysis reveals that Pseudomonas aeruginosa virulence is combinatorial. *Genome Biol.* **7**, 1–14 (2006).

87. Cady, K. C., Bondy-Denomy, J., Heussler, G. E., Davidson, A. R. & O'Toole, G. A. The CRISPR/Cas adaptive immune system of Pseudomonas aeruginosa mediates resistance to naturally occurring and engineered phages. *J. Bacteriol.* **194**, 5728–5738 (2012).

88. Cady, K. C. *et al.* Prevalence, conservation and functional analysis of Yersinia and Escherichia CRISPR regions in clinical Pseudomonas aeruginosa isolates. *Microbiology* **157**, 430–437 (2011).

89. Winsor, G. L. *et al.* Pseudomonas Genome Database: facilitating user-friendly, comprehensive comparisons of microbial genomes. *Nucleic Acids Res.* **37**, D483–8 (2009).

90. Marino, N. D. *et al.* Discovery of widespread type I and type V CRISPR-Cas inhibitors. *Science* **362**, 240–242 (2018).

91. Blower, T. R., Evans, T. J., Przybilski, R., Fineran, P. C. & Salmond, G. P. C. Viral evasion of a bacterial suicide system by RNA-based molecular mimicry enables infectious altruism. *PLoS Genet.* **8**, e1003023 (2012).

92. Qiu, D., Damron, F. H., Mima, T., Schweizer, H. P. & Yu, H. D. PBAD-based shuttle vectors for functional analysis of toxic and highly regulated genes in Pseudomonas and Burkholderia spp. and other bacteria. *Appl. Environ. Microbiol.* **74**, 7422–7426 (2008).
