# Supplementary material for: Bacteriophages suppress CRISPR–Cas immunity using RNA-based anti-CRISPRs
Source: Nature. 2023 Oct 18;623(7987):601–7. doi: 10.1038/s41586-023-06612-5 (PMC10651486; doi:10.1038/s41586-023-06612-5)
Supplement: Supplementary file 2 — Reporting Summary [file 41586_2023_6612_MOESM2_ESM.pdf]

nature portfolio

Corresponding author(s): Rafael Pinilla-Redondo
Peter C. Fineran

Last updated by author(s): August 28th, 2023

Reporting Summary

Nature Portfolio wishes to improve the reproducibility of the work that we publish. This form provides structure for consistency and transparency in reporting. For further information on Nature Portfolio policies, see our Editorial Policies and the Editorial Policy Checklist.

Statistics

For all statistical analyses, confirm that the following items are present in the figure legend, table legend, main text, or Methods section.

n/a	Confirmed	
☐	☒	The exact sample size (*n*) for each experimental group/condition, given as a discrete number and unit of measurement
☐	☒	A statement on whether measurements were taken from distinct samples or whether the same sample was measured repeatedly
☐	☒	The statistical test(s) used AND whether they are one- or two-sided<br>*Only common tests should be described solely by name; describe more complex techniques in the Methods section.*
☒	☐	A description of all covariates tested
☒	☐	A description of any assumptions or corrections, such as tests of normality and adjustment for multiple comparisons
☐	☒	A full description of the statistical parameters including central tendency (e.g. means) or other basic estimates (e.g. regression coefficient) AND variation (e.g. standard deviation) or associated estimates of uncertainty (e.g. confidence intervals)
☐	☒	For null hypothesis testing, the test statistic (e.g. *F*, *t*, *r*) with confidence intervals, effect sizes, degrees of freedom and *P* value noted<br>*Give P values as exact values whenever suitable.*
☒	☐	For Bayesian analysis, information on the choice of priors and Markov chain Monte Carlo settings
☒	☐	For hierarchical and complex designs, identification of the appropriate level for tests and full reporting of outcomes
☒	☐	Estimates of effect sizes (e.g. Cohen's *d*, Pearson's *r*), indicating how they were calculated

Our web collection on statistics for biologists contains articles on many of the points above.

Software and code

Policy information about availability of computer code

Data collection	CCtyper 1.2.1; CCTyper Web Server (December 2020); SRUFinder (v0.2.1)
Data analysis	cd-hit-env; prodigal; fetchMGs 1.2; mafft 7.310; Fasttree 2.1.10; R; iTOL v. 5; CCtyper 1.2.1; CCTyper Web Server (December 2020); tblastn 2.11.0+; FACSDiva software (v.8, BD Biosciences); FlowJo software v.10; Bowtie 2; SAMtools; RNAfold v. 2.4.9, RNA2Drawer v. 6.3; BD FACSDiva™ Software v.8; FlowJo Software v.10.8.1; FastQC v. 0.11.9; Samtools v. 1.16.1, Geneious Prime® 2022.1.1; Prism v.9.2.0; Prodigal 2.6.3; R version 4.1; Trimmomatic v 0.39. The algorithm of SRUfinder is available as a python package (https://pypi.org/project/srufinder) which is also a conda package (https://anaconda.org/russel88/srufinder). The custom code was deposited in Zenedo: Russel88/SRUFinder (v0.2.1), https://doi.org/10.5281/zenodo.8273406(Russel 2023).

For manuscripts utilizing custom algorithms or software that are central to the research but not yet described in published literature, software must be made available to editors and reviewers. We strongly encourage code deposition in a community repository (e.g. GitHub). See the Nature Portfolio guidelines for submitting code & software for further information.

Data

Policy information about availability of data

All manuscripts must include a data availability statement. This statement should provide the following information, where applicable:

- Accession codes, unique identifiers, or web links for publicly available datasets
- A description of any restrictions on data availability
- For clinical datasets or third party data, please ensure that the statement adheres to our policy

Data that support the findings of this study are available within the article and its Supplemtary tables and data. Small RNA sequencing is BioProject accession PRJNA893428 and BioSample accession SAMN31422748 (https://www.ncbi.nlm.nih.gov/bioproject/893428). We used the following datasets: PLSDB plasmid database (2020_11_19), IMG/VR3 database, and PHASTER database. The database of 17,823 non-redundant CRISPR repeat sequences with known associated subtypes was made available here: https://github.com/Russel88/SRUFinder/blob/master/data/repeats.fa

Research involving human participants, their data, or biological material

Policy information about studies with human participants or human data. See also policy information about sex, gender (identity/presentation), and sexual orientation and race, ethnicity and racism.

Reporting on sex and gender	N.A.
Reporting on race, ethnicity, or other socially relevant groupings	N.A.
Population characteristics	N.A.
Recruitment	N.A.
Ethics oversight	N.A.

Note that full information on the approval of the study protocol must also be provided in the manuscript.

Field-specific reporting

Please select the one below that is the best fit for your research. If you are not sure, read the appropriate sections before making your selection.

☒ Life sciences ☐ Behavioural & social sciences ☐ Ecological, evolutionary & environmental sciences

For a reference copy of the document with all sections, see nature.com/documents/nr-reporting-summary-flat.pdf

Life sciences study design

All studies must disclose on these points even when the disclosure is negative.

Sample size	At least three random colonies were picked as biological replication for each assay. The number of selected colonies (our sample size) were determined based on standards in the field for these types of experiments.
Data exclusions	No data was excluded from the analysis
Replication	All of the measurements were performed in at least three biological replicates, and attempts at replication were successful. The methods and reagents used are described in detail to ensure reproducibility.
Randomization	Random colonies of transformants were picked as biological replicates. Other aspects of randomization are not relevant (e.g., covariates) for the experimental designs presented in this work. This work did not require randomization due to human or animal subjects.
Blinding	No blinding was performed, as the work did not involve human or animal subjects. Blinding to sample group allocation is not typically relevant to bacterial and biochemical analyses.

Reporting for specific materials, systems and methods

We require information from authors about some types of materials, experimental systems and methods used in many studies. Here, indicate whether each material, system or method listed is relevant to your study. If you are not sure if a list item applies to your research, read the appropriate section before selecting a response.

Materials & experimental systems

n/a	Involved in the study	
☒	☐	Antibodies
☒	☐	Eukaryotic cell lines
☒	☐	Palaeontology and archaeology
☒	☐	Animals and other organisms
☒	☐	Clinical data
☒	☐	Dual use research of concern
☒	☐	Plants

Methods

n/a	Involved in the study	
☒	☐	ChIP-seq
☐	☒	Flow cytometry
☒	☐	MRI-based neuroimaging

Flow Cytometry

Plots

Confirm that:

☒ The axis labels state the marker and fluorochrome used (e.g. CD4-FITC).

☒ The axis scales are clearly visible. Include numbers along axes only for bottom left plot of group (a 'group' is an analysis of identical markers).

☒ All plots are contour plots with outliers or pseudocolor plots.

☒ A numerical value for number of cells or percentage (with statistics) is provided.

Methodology

Sample preparation	Bacterial cells from glyverol stock were diluted in 1 mL of phosphate-buffered saline (PBS, 1:1000) and kept on ice until analysis on the flow cytometer.
Instrument	BD LSRFortessa™ Cell Analyzer
Software	BD FACSDiva™ Software v.8 and FlowJo Software v.10.8.1
Cell population abundance	20,000 events were recorded per sample
Gating strategy	Cells were gated on SSC-A/SSC-H and SSC-A/FSC-A, then bifurcated (using BifurGate) into mCherry+ and mCherry– populations. The mCherry was excited using a yellow–green laser (561 nm) and detected with a 610/20 nm bandpass filter.

☒ Tick this box to confirm that a figure exemplifying the gating strategy is provided in the Supplementary Information.
